# Substituted Purines as High-Affinity Histamine H_3_ Receptor Ligands

**DOI:** 10.3390/ph15050573

**Published:** 2022-05-04

**Authors:** Christian Espinosa-Bustos, Luisa Leitzbach, Tito Añazco, María J. Silva, Andrea del Campo, Alejandro Castro-Alvarez, Holger Stark, Cristian O. Salas

**Affiliations:** 1 Departamento de Farmacia, Facultad de Química y de Farmacia, Pontificia Universidad Católica de Chile, Santiago 7820436, Chile; tanazco@uc.cl (T.A.); mjsilva15@uc.cl (M.J.S.); andrea.delcampo@uc.cl (A.d.C.); 2 Institute of Pharmaceutical and Medicinal Chemistry, Heinrich Heine University Düsseldorf, Universitaetsstr. 1, 40225 Duesseldorf, Germany; luisa.leitzbach@hhu.de (L.L.); stark@hhu.de (H.S.); 3 Laboratorio de Bioproductos Farmacéuticos y Cosméticos, Centro de Excelencia en Medicina Traslacional, Facultad de Medicina, Universidad de La Frontera, Temuco 4780000, Chile; qf.alec.astro@gmail.com; 4 Departamento de Química Orgánica, Facultad de Química y de Farmacia, Pontificia Universidad Católica de Chile, Santiago 7820436, Chile

**Keywords:** histamine H_3_ receptor, H_3_R ligands, purines, in silico studies, ADME prediction

## Abstract

Continuing with our program to obtain new histamine H_3_ receptor (H_3_R) ligands, in this work we present the synthesis, H_3_R affinity and in silico studies of a series of eight new synthetically accessible purine derivatives. These compounds are designed from the isosteric replacement of the scaffold presented in our previous ligand, pyrrolo[2,3-*d*]pyrimidine ring, by a purine core. This design also considers maintaining the fragment of bipiperidine at C-4 and aromatic rings with electron-withdrawing groups at N-9, as these fragments are part of the proposed pharmacophore. The in vitro screening results show that two purine derivatives, **3d** and **3h**, elicit high affinities to the H_3_R (Ki values of 2.91 and 5.51 nM, respectively). Both compounds are more potent than the reference drug pitolisant (K_i_ 6.09 nM) and show low toxicity with in vitro models (IC_50_ > 30 µM on HEK-293, SH-SY5Y and HepG2 cell lines). Subsequently, binding modes of these ligands are obtained using a model of H_3_R by docking and molecular dynamics studies, thus determining the importance of the purine ring in enhancing affinity due to the hydrogen bonding of Tyr374 to the N-7 of this heterocycle. Finally, in silico ADME properties are predicted, which indicate a promising future for these molecules in terms of their physical–chemical properties, absorption, oral bioavailability and penetration in the CNS.

## 1. Introduction

**Figure 1:**
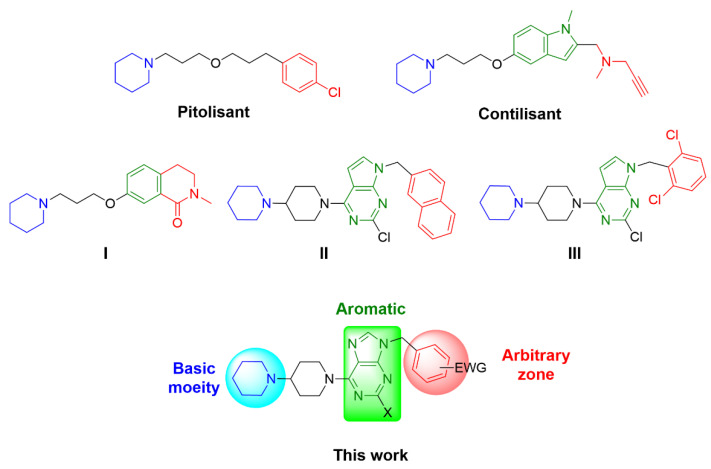
Chemical structures of H_3_R ligands containing piperidine moiety, and the proposed structures in this work.

The histamine 3 receptor (H_3_R) belongs to a superfamily of G protein-coupled receptors (GPCRs) and has received great interest in recent years due to its distribution, mainly in the central nervous system, acting as an auto- and heteroreceptor and regulating the release of histamine itself and other neurotransmitters, such as acetylcholine and dopamine, among others [1,2,3]. Antagonists or inverse agonists of this receptor prevent the activation of the H_3_R and therefore, due to negative feedback, increase the concentration of histamine and the other neurotransmitters mentioned above. Therefore, these are being investigated as therapeutic alternatives in neurodegenerative diseases such as Alzheimer’s and Parkinson’s, as well as in disorders such as narcolepsy and attention deficit hyperactivity disorder. In present times, the H_3_R inverse agonist pitolisant (Wakix, Figure 1) is the only drug approved by the EMA for the treatment of narcolepsy with or without cataplexy and for obstructive sleep apnea, which has increased interest in the clinical applications of novel H_3_R inverse agonists or antagonists [4,5].

In this regard, many series of compounds with great structural diversity have been synthesized [6]. However, there is a common pharmacophore consisting of a basic moiety, separated by a linker (often saturated alkyl chains) from an aromatic zone attached to an arbitrary scaffold that is generally hydrophobic [7]. First generation H_3_R ligands usually contain imidazole-based scaffolds, with regard to histamine structure [8]. Otherwise, the second generation of H_3_R ligands, the imidazole heterocycle, is replaced by piperidine moieties [9].

In recent years, several H_3_R ligands that contain the piperidine heterocycle in their structure have been reported. Contilisant (Figure 1) was reported to display H_3_R antagonism as well as cholinesterase and MAO-B inhibition [10]. Zhang et al. published a series of lactams that contain several basic scaffolds in their structure, in which compound **I** shows great affinity for the H_3_R and selectivity against the H_1_R [11]. On the other hand, our research group has reported several pyrrolo[2,3-*d*]pyrimidines substituted in position C-4 with the bipiperidine moiety, obtaining affinities for H_3_R in the nanomolar range and highlighting compounds **II** and **III**, which show K_i_ values of 126 and 6.0 nM, respectively [12,13]. Our previous antecedents suggest that derivatives with a higher affinity for H_3_Rs are obtained using bipiperidine as a basic moiety, whereas in the arbitrary zone, the best results have used aromatic rings with electron-withdrawing groups (EWG).

In order to improve affinity to the H_3_R, in this work we propose the use of purine heterocycle as a pyrrolo[2,3-*d*]pyrimidine bioisostere while maintaining the aforementioned substitution patterns present in **III** (Figure 1). Purine heterocycle is selected because this scaffold is part of several compounds with diverse biological activities [14,15,16,17]. Moreover, this heterocycle significantly improves pharmacological activities and physicochemical properties [18]. In addition, the effects of EWG on the aromatic ring at N-9 are considered as well as the replacement of a hydrogen atom at the C-2 position by a chlorine atom. Later, the synthesized purine derivatives are evaluated for their affinity to the human H_3_R and the most promising compounds are evaluated for their cytotoxicity on cell lines. Finally, docking and molecular dynamic studies are carried out to understand the effects of chemical modifications on affinity to the H_3_R. Considering the great need to find new therapeutic alternatives for the treatment of neurodegenerative diseases, this work contributes to medicinal chemistry through the discovery of easily accessible compounds with high affinity to the H_3_R, with low toxicity in the cell lines evaluated and with a promising ADME profile.

## 2. Results and Discussion

### 2.1. Chemistry

**Scheme 1:**
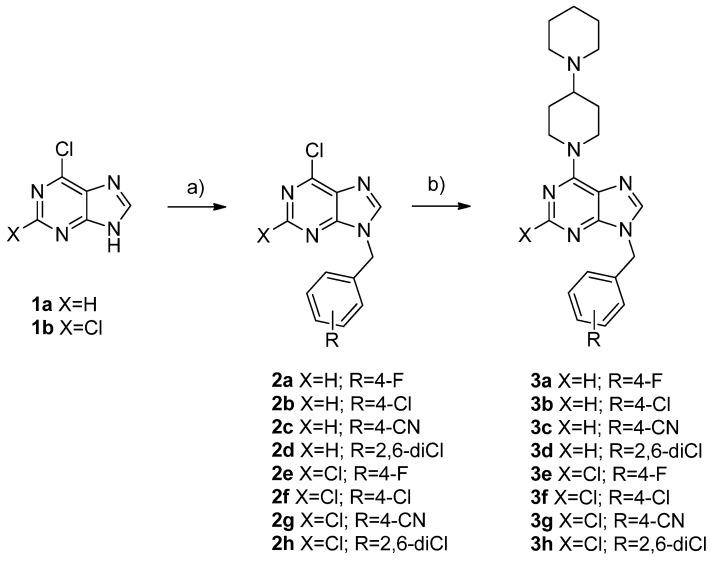
Synthesis of purine derivatives **3a–h**. Reagents and conditions: (a) Benzyl halides, K_2_CO_3_, CH_3_CN, reflux, 2–4 h, 19–48%. (b) Bipiperidine, DIPEA, ethanol, reflux, 1–2 h, 41–66%.

The general synthetic procedures to obtain 6-([1,4’-bipiperidin]-1’-yl)-9-aryl-9*H*-purines **3a–d** and 6-([1,4’-bipiperidin]-1’-yl)-9-aryl-2-halo-9*H*-purines **3e–h** are summarized in Scheme 1. The derivatives **2a–h** were synthesized by nucleophilic substitution among the commercially available 6-chloro-9*H*-purine derivatives **1a–b** and the respective benzyl halide, in low to moderate yields. In this first step, a mixture of N-9 and N-7-alkylated purines were obtained in a proportion of 4:1, respectively. In a second step, a nucleophilic substitution (S_N_Ar) reaction was achieved with N-9 substituted purines, using bipiperidine, providing the target compounds **3a–h**, with good yields (>60%). The chemical structures of the purine derivatives were established based on their spectral properties (IR, ^1^H NMR, ^13^C NMR and HRMS, see experimental section and Supplementary Materials).

### 2.2. H_3_R Affinity

**Figure 2:**
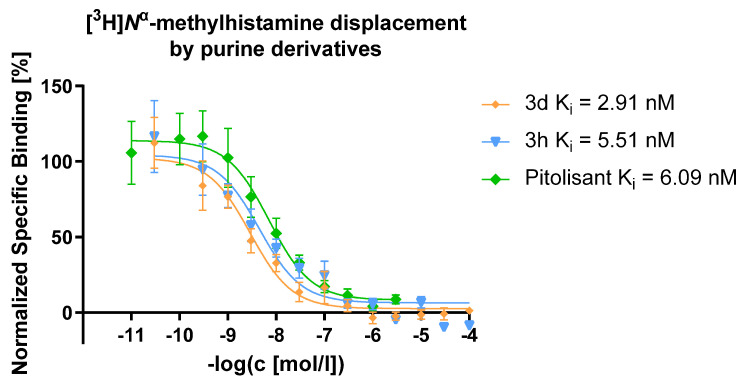
Displacement of [^3^H]*N^α^*-methylhistamine (c = 2 nM, K_d_ = 3.08 nM by **3d** (K_i_ = 2.91 nM), **3h** (K_i_ = 5.51 nM) and pitolisant (K_i_ = 6.09 nM). The assay was performed on HEK-293 cell membranes stably expressing the human H_3_R. Data are expressed as mean values ± SEM (at least three independent experiments, each performed in duplicate).

Compounds **3a–h** were screened for their affinities to the human H_3_R by performing competitive radioligand binding assays using [^3^H]*N*^α^-methylhistamine as a radioligand (Table 1). All compounds presented K_i_ values in the nanomolar range, confirming that the purine heterocycle provides derivatives with a high affinity to the receptor and that some substitution patterns are important in structure–activity relationships. Firstly, the presence of a chlorine atom at the C-2 position maintains or slightly decreases affinity to H_3_R, as observed in **3a–d** versus **3e–h** compounds. In the arbitrary zone, the presence of the *p*-fluorophenyl portion yields the derivatives with the lowest affinity (**3a** and **3e**), and this is an interesting result that correlates with our previous works [13]. Finally, the substitution in N-9 with 2,6-dichlorobenzyl provides the most active derivatives of this series, **3d** and **3h**, which present K_i_ values of 2.91 nM and 5.51 nM, respectively, being even better than those presented by pitolisant (Figure 2). In this case, a purine scaffold appears to increase affinity to H_3_R compared to derivatives with pyrrolo[2,3-*d*]pyrimidine [13], but this observation is not decisive, since this tendency is not observed in other derivatives.

### 2.3. Cytotoxicity Assays

The potential general nephrotoxic, neurotoxic and hepatotoxic effects of selected H_3_R ligands **3d** and **3h** were investigated to determine their influence on the viability of normal HEK-293, neuroblastoma SH-SY5Y and hepatoma HepG2 cell lines by a colorimetric MTT reduction assay (Table 2). Tested compounds showed similar and weak influences on the viability of all examined cell lines, which may indicate that both compounds would not be potentially cytotoxic in future in vivo studies.

### 2.4. Docking Studies

Although the crystal structure of the H_3_R receptor is not yet available, the inactive H_3_R model was downloaded from the GPCRdb website (https://gpcrdb.org, accessed on 1 November 2021), which comes from homology modelling using the crystal of the histamine H_1_ receptor (PDB ID: 3RZE) as a template [19]. Subsequently, an energetic minimization of the residues was performed to adjust the dihedral angles of each amino acid. According to the literature, the critical residues of the orthosteric binding pocket are Asp114 and Glu206. Both residues assist in recognizing the endogenous ligand (Figure S1), forming salt bridges of the carboxylates of these residues with the cationic amines of the synthesized ligands [20].

**Figure 3:**
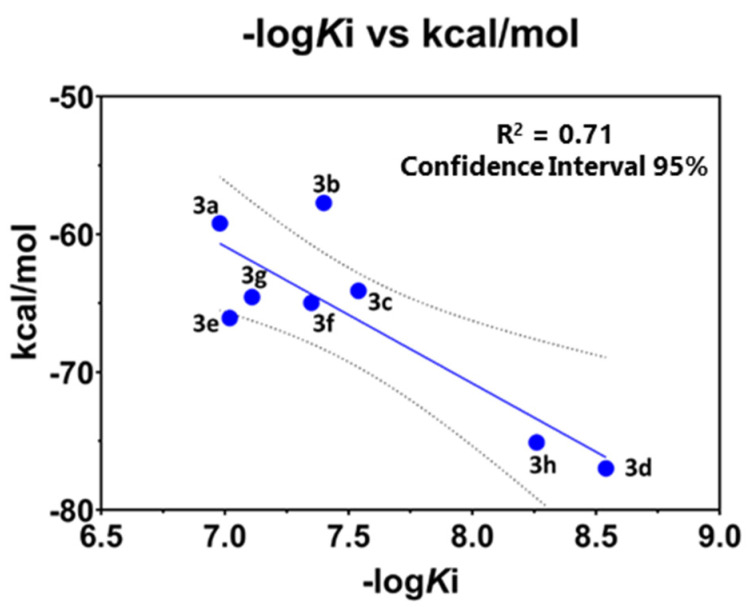
Correlation graph for binding free energy predicted activity (kcal/mol) and -logK_i_ values.

Molecular docking studies were carried out to understand the binding mode of the most active compound, **3d**, within the H_3_R binding site. A standard protocol was used to perform induced-fit docking, which generated four binding poses for **3d**. The four best poses were analyzed based on their docking scores and polar and non-polar interactions. The best poses showed docking scores of −10.23 kcal/mol with interaction with Asp114 and of −9.18 kcal/mol with Glu206 (Figure S2). Therefore, the lowest energy pose was selected, and the other synthesized compounds were screened. Once the pose was obtained for each ligand, an MM-GBSA calculation was performed, energetically minimizing the residues composing the binding site at about 6 Å from the center of mass of the ligands. Finally, the binding free energy was calculated for each of them (Table 3). With an R^2^ of 0.71, the correlation between the estimated affinity energy and the experimental data (-logK_i_) was reached, indicating that the resulting posture is the best representative of biological activity and affinity with this receptor (Figure 3).

**Figure 4:**
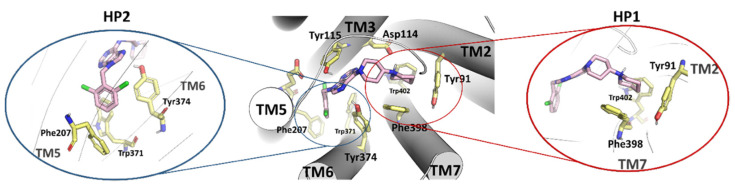
Binding mode of **3d** in the H_3_R model.

Ligand–receptor interactions can be divided into three regions constituting the synthesized ligands (Figure 4), where the basic moiety interacts firstly with a hydrophobic pocket (hydrophobic pocket 1, HP1) composed of three aromatic rings of residues Tyr91, Phe398 and Trp402. In addition, there is an electrostatic interaction between the cationic amine of piperidine with Asp114. Next, the aromatic region of purine forms a π-stacking interaction with Tyr115 and a hydrogen bond with the N-7 of purine core with the hydroxyl of Tyr374. Finally, the halogenated aromatic region interacts with a second hydrophobic pocket (HP2) consisting of Tyr374, Trp371, Phe207 and Phe367. This region interacts favorably with the electron-deficient ring of the synthesized ligands, and halogen substitution has a direct impact on the favorable hydrophobic interaction, with the *para*-position being the least favorable due to a steric hindrance it causes with Phe207, which coincides with substitutions in the *ortho-*position being the most active, as is the case for compounds **3d** and **3h**.

**Figure 5:**
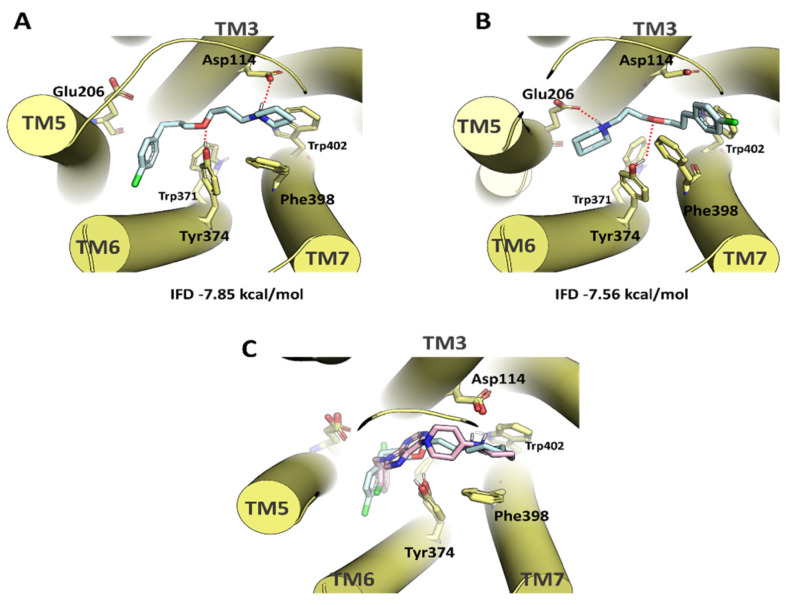
(**A**) The best pose of pitolisant interacting with Asp114; (**B**) The best pose of pitolisant interacting with Glu206; (**C**) The overlap between the best pose achieved for **3d** and pitolisant.

The same protocol was repeated to obtain the best poses with pitolisant, considering the interaction of protonated piperidine with Asp114 and Glu206. However, the difference in affinity energy between both poses was not so vast (Figure 5). Additionally, apart from an electrostatic interaction of the carboxylate with Asp114 or Glu206 (regardless of what the case may be), the ether oxygen interacts, forming a hydrogen bond with Tyr374, thus highlighting the importance of this residue for the affinity for the binding site.

### 2.5. Molecular Dynamic Studies

To understand the affinity differences between the most active compound and the less active compound, molecular dynamics of 50 ns were performed with the ligand–receptor complexes of compound **3d** (most active compound) and **3a** (least active compound). We considered the phospholipids that constitute the lipid membrane and a solvated system (water), and we obtained a transmembrane system.

**Figure 6:**
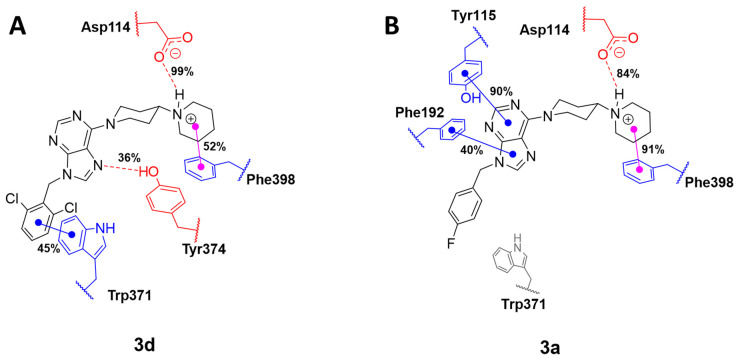
Binding modes of **3d** (**A**) and **3a** (**B**) during the 50 ns MD simulations at H_3_R. Red dotted lines represent hydrogen bonds, blue lines represent hydrophobic interactions and violet lines represent π-cation interactions. Percentages represent specific interactions during the simulation.

The trajectories were analyzed with the Simulation Interaction Diagram module of the Schrodinger Suite. The ligand–protein interactions revealed the residues that persist the most during molecular dynamics. The **H_3_R–3d** complex interacts with Asp114 with almost 99% during the whole trajectory, with an average distance of 2.06 Å between the amine proton of the piperidine and the carboxylate, whereas **3a** has an interaction with 88% of the molecular dynamics with an average distance of 2.22 Å (Figure 6). Another important aspect is the presence of hydrogen bonding of Tyr374 with the N-7 of purine in compound **3d** with a presence of 36%, which, in turn, caused an enhanced π-stacking interaction with Trp371 with 45% during molecular dynamics.

The molecular dynamics of the **H_3_R–3a** system demonstrates the importance of the halogen atom in the *para* position of the aromatic ring and its low biological activity, which responds to the steric hindrance generated by the halogen with the Phe207 residue, causing a very poor interaction with Tyr374 and Trp371 but improving hydrophobic interactions with Tyr115 and Phe192.

Considering the molecular dynamics results, both systems, **H_3_R–3d** and **H_3_R–3a**, reveal positive interactions at the binding site. However, **3d** interacts preferentially with TM6 domain residues (Trp371 and Tyr374). In contrast, **3a** interacts with Tyr115, a residue belonging to TM3, and with Phe192, an amino acid residue belonging to the extracellular loop (Figure 6). Therefore, the inverse agonist action of the most active compound (**3d**) may be explained by the interaction between the TM3 (Asp114) and TM6 (Trp371 and Tyr374) domains, similar to that which occurs with other synthesized ligands that have a similar mechanism of action [21,22].

### 2.6. In Silico ADME and Drug-Likeness Properties

**Figure 7:**
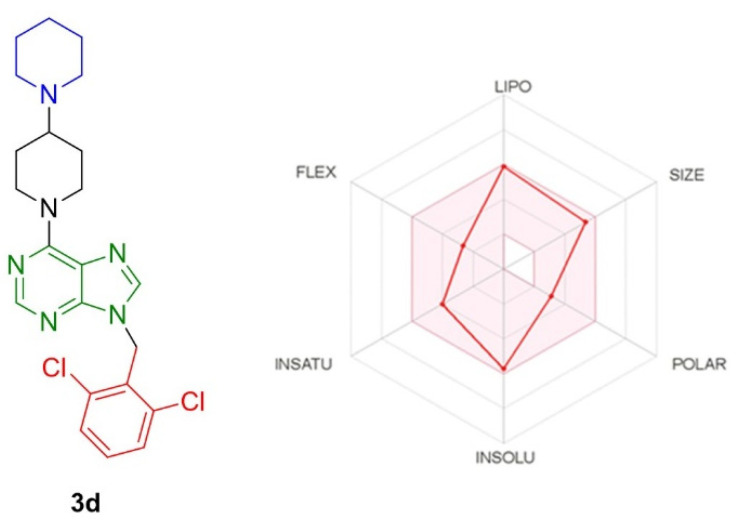
The bioavailability radar chart of **3d**. The pink area indicates the range of the values of the optimal oral bioavailability property, and the predicted properties are shown as red lines. The radar was generated by SwissADME (http://www.swissadme.ch/index.php, accessed on 30 March 2021).

Employing the SwissADME online webserver [23], compounds **3a–h** were analyzed to predict their physicochemical properties, in relation to absorption, bioavailability and blood–brain barrier (BBB) permeability. Lipinski’s rule establishes that an orally bioavailable drug should not violate the following criteria: ≤5 hydrogen bond donors (HBD); ≤10 hydrogen bond acceptors (HBA); a MW of <500 g/mol; and a log *p* value of <5 [24]. Moreover, Veber et al. described the magnitude of PSA and the number of rotatable bonds as criteria to rate oral bioavailability. Veber’s rule states that, to be orally bioavailable, a molecule should have either a PSA ≤ 140 Å and ≤10 rotatable bonds (NRB) or ≤12 HBD and HBA in total and ≤10 rotatable bonds [25]. As shown in Table 4, compounds **3a–h** accomplished the drug-likeness criteria described by Lipinski and Veber; hence, they are expected to have good oral bioavailability. Moreover, according to the brain or intestinal estimated permeation method (BOILED-Egg), all compounds should have good gastrointestinal absorption and should cross the blood–brain barrier [26], and this is an important result considering the CNS distribution of the H_3_R. Furthermore, the SwissADME server resolved that compounds **3a–h** should have no pan-assay interference liability (PAINS). In addition, the SwissADME server indicated that the bioavailability radar chart for **3d** was within the desired range (pink region) for six parameters used for oral absorption prediction: flexibility (FLEX), lipophilicity (LIPO), solubility (INSOLU), size and polarity (POLAR) and saturation (INSATU) (Figure 7), which can foresee their predicted good oral bioavailability.

## 3. Materials and Methods

### 3.1. Chemistry

Melting points were determined on a Thermogeräte Kofler apparatus (Reichert, Werke A.G., Wien, Austria) and were uncorrected. All reagents were purchased from Sigma-Aldrich (St. Louis, MO, USA) unless otherwise specified. The NMR spectra were recorded at NMR Bruker AV 400 or 200 MHz. Chemical shifts were given in parts per million relative to TMS [^1^H and ^13^C, δ (SiMe_4_) = 0]. Most NMR assignments were supported by additional 2D experiments. HRMS-ESI-MS experiments were carried out using a high-resolution mass spectrometer, Exactive™ Plus Orbitrap, ThermoFisher Scientific (Bremen, Germany). Thin layer chromatography (TLC) was performed using Merck GF-254 type 60 silica gel. Column chromatography was carried out using Merck silica gel 60 (70–230 mesh). Compound purity ≥ 95% was determined by HPLC Shimadzu 10-ADvp (Shimadzu, Kioto, Japón) with a C18 column (Kinetex 5u XB-C18), an acetonitrile/water (0.1% formic acid) mobile phase and a flow rate of 1 mL/min for 15 min.

#### 3.1.1. General Procedure for the Synthesis of **2a–h** Derivatives

A mixture of purines **1a-b** (1.0 mmol), the respective benzyl halide (1.2 mmol) and potassium carbonate (3.0 mmol) in acetonitrile (5 mL) were stirred for 3 h, and then the mixture was filtered and evaporated under vacuum. The products were separated by flash chromatography on silica gel eluted with dichloromethane.

6-Chloro-9-(4-fluorobenzyl)-9*H*-purine **2a**. White solid, yield 37%, m.p. 135–138 °C. (lit. 129–131 °C) [27]. ^1^H NMR (400 MHz, CDCl_3_) δ 8.95 (s, 1H), 8.30 (s, 1H), 7.51 (dd, *J* = 8.5, 5.2 Hz, 2H), 7.23 (t, *J* = 8.5 Hz, 2H), 5.61 (s, 2H). ^13^C NMR (101 MHz, CDCl_3_) δ 164.10–161.63 (d, ^1^*J_CF_* = 248.6 Hz), 152.23, 151.77, 151.20, 144.78, 131.49, 130.44–130.41 (d, ^4^*J_CF_* = 3.4 Hz), 129.97–129.89 (d, ^3^*J_CF_* = 8.4 Hz), 116.40–116.18 (d, ^2^*J_CF_* = 21.8 Hz), 47.24. ^19^F NMR (376 MHz, CDCl_3_) δ −112.32 (1F).

6-Chloro-9-(4-chlorobenzyl)-9*H*-purine **2b**. White solid, yield 34%, m.p. 134–136 °C. (lit. 130–133 °C) [27]. ^1^H NMR (200 MHz, CDCl_3_) δ 8.77 (s, 1H), 8.14 (s, 1H), 7.30 (q, *J* = 8.5 Hz, 4H), 5.43 (s, 2H). ^13^C NMR (50 MHz, CDCl_3_) δ 152.27, 151.74, 151.23, 144.77, 134.94, 133.00, 131.42, 129.46 (2C), 129.32 (2C), 47.25.

4-((6-Chloro-9*H*-purin-9-yl)methyl)benzonitrile **2c**. White solid, yield 38%, m.p. 205–207 °C. ^1^H NMR (200 MHz, CDCl_3_) δ 8.77 (s, 1H), 8.18 (s, 1H), 7.67 (d, *J* = 7.8 Hz, 2H), 7.42 (d, *J* = 8.0 Hz, 2H), 5.54 (s, 2H). ^13^C NMR (50 MHz, CDCl_3_) δ 152.44, 151.75, 151.51, 144.71, 139.66, 133.02 (2C), 131.48, 128.41 (2C), 117.97, 112.95, 47.34.

6-Chloro-9-(2,6-dichlorobenzyl)-9*H*-purine **2d**. White solid, yield 36%, m.p. 188–191 °C. (lit. 186–188 °C) [27]. ^1^H NMR (400 MHz, CDCl_3_) δ 8.79 (s, 1H), 7.93 (s, 1H), 7.43 (d, *J* = 8.2 Hz, 2H), 7.38–7.29 (m, 1H), 5.73 (s, 2H). ^13^C NMR (101 MHz, CDCl_3_) δ 152.11, 151.81, 151.06, 144.31, 136.78, 131.37, 131.26, 129.87, 129.06 (3C), 43.13.

2,6-Dichloro-9-(4-fluorobenzyl)-9*H*-purine **2e**. White solid, yield 43%, m.p. 133–136 °C. (lit. 120–122 °C) [27]. ^1^H NMR (200 MHz, CDCl_3_) δ 8.07 (s, 1H), 7.33 (dd, *J* = 8.3, 5.2 Hz, 2H), 7.08 (t, *J* = 8.4 Hz, 2H), 5.39 (s, 2H). ^13^C NMR (50 MHz, CDCl_3_) δ 165.44–160.49 (d, ^1^*J_CF_* = 249.0 Hz), 153.26, 153.06, 151.96, 145.35, 130.66, 130.16–129.99 (d, ^3^*J_CF_* = 8.4 Hz, 2C), 129.93–129.87 (d, ^4^*J_CF_* = 3.4 Hz), 116.63–116.20 (d, ^2^*J_CF_* = 21.9 Hz, 2C), 47.36.

2,6-Dichloro-9-(4-chlorobenzyl)-9*H*-purine **2f**. White solid, yield 48%, m.p. 158–160 °C (lit. 162–164 °C) [27]. ^1^H NMR (200 MHz, CDCl_3_) δ 8.08 (s, 1H), 7.36 (d, *J* = 8.5 Hz, 2H), 7.26 (d, *J* = 8.5 Hz, 2H), 5.39 (s, 2H). ^13^C NMR (50 MHz, CDCl_3_) δ 153.31, 153.06, 152.00, 145.31, 135.17, 132.49, 130.62, 129.58 (2C), 129.43 (2C), 47.35.

4-((2,6-Dichloro-9*H*-purin-9-yl)methyl)benzonitrile **2g**. White solid, yield 19%, m.p. 199–202 °C. ^1^H NMR (200 MHz, CDCl_3_) δ 8.12 (s, 1H), 7.70 (d, *J* = 7.9 Hz, 2H), 7.42 (d, *J* = 8.0 Hz, 2H), 5.50 (s, 2H). ^13^C NMR (50 MHz, CDCl_3_) δ 153.59, 153.05, 152.36, 145.18, 139.11, 133.13 (2C), 130.65, 128.47 (2C), 117.88, 113.21, 47.39.

2,6-Dichloro-9-(2,6-dichlorobenzyl)-9*H*-purine **2h**. White solid, yield 22%, m.p. 183–186 °C. ^1^H NMR (200 MHz, CDCl_3_) δ 7.89 (s, 1H), 7.54–7.30 (m, 3H), 5.70 (s, 2H). ^13^C NMR (50 MHz, CDCl_3_) δ 153.11, 151.77, 144.82, 136.79, 131.56 (2C), 130.43, 129.37, 129.10 (3C), 43.34.

#### 3.1.2. General Synthetic Procedure to Obtain Substituted Purines **3a–h**

The *N*-benzyl-purines **2a–h** (1.0 mmol), 4-piperidinopiperidine (2.0 mmol), triethylamine (3.0 mmol) and ethanol (5 mL) were added to a microwave reaction flask, and the reaction mixture was irradiated for 15 min at 80 °C. Then, the solvent was evaporated under vacuum, and the crude product was purified by column chromatographic on silica gel using a (8:2) chloroform/methanol mixture.

6-([1,4’-Bipiperidin]-1’-yl)-9-(4-fluorobenzyl)-9*H*-purine **3a**. White solid, yield 49%, m.p. 138–139 °C. ^1^H NMR (400 MHz, CDCl_3_) δ 8.57 (s, 1H), 7.90 (s, 1H), 7.47 (t, *J* = 6.4 Hz, 2H), 7.23 (t, *J* = 8.4 Hz, 2H), 5.76 (bs, 2H), 5.53 (s, 2H), 3.25 (t, *J* = 12.3 Hz, 2H), 2.93 (t, *J* = 10.9 Hz, 1H), 2.81 (s, 4H), 2.25 (d, *J* = 12.1 Hz, 2H), 1.95–1.77 (m, 6H), 1.67 (bs, 2H). ^13^C NMR (101 MHz, CDCl_3_) δ 163.81–161.35 (d, ^1^*J_CF_* = 247.2 Hz), 153.75, 152.71, 150.94, 137.88, 131.73–131.69 (d, ^4^*J_CF_* = 3.2 Hz), 129.55–129.47 (d, ^3^*J_CF_* = 8.3 Hz), 119.79, 116.05–115.83 (d, ^2^*J_CF_* = 21.7 Hz), 63.03, 50.12, 46.33, 44.91, 27.91, 25.83, 24.43. ^19^F NMR (376 MHz, CDCl_3_) δ −113.57 (1F). HRMS calcd. for (C_22_H_27_FN_6_ [M + H]^+^): 395.2354. Found: 395.2350. HPLC purity: 99.97%.

6-([1,4’-Bipiperidin]-1’-yl)-9-(4-chlorobenzyl)-9*H*-purine **3b**. White solid, yield 55%, m.p. 156–157 °C. ^1^H NMR (400 MHz, CDCl_3_) δ 8.30 (s, 1H), 7.65 (s, 1H), 7.19 (d, *J* = 29.2 Hz, 4H), 5.48 (s, 2H), 5.26 (s, 2H), 2.98 (b.s, 2H), 2.52 (b.s, 5H), 1.96 (b.s, 2H), 1.73–1.40 (m, 8H). ^13^C NMR (101 MHz, CDCl_3_) δ 153.73, 152.72, 150.92, 137.83, 134.39, 129.13 (2C), 128.98 (2C), 119.73, 62.92, 50.12, 46.31, 44.99, 27.99, 25.99, 24.52. HRMS calcd. for (C_22_H_27_ClN_6_ [M + H]^+^): 411.2058. Found: 411.2057. HPLC purity: 99.39%.

4-((6-([1,4’-Bipiperidin]-1’-yl)-9*H*-purin-9-yl)methyl)benzonitrile **3c**. White solid, yield 41%, m.p. 136–138 °C. ^1^H NMR (400 MHz, CDCl_3_) δ 8.29 (s, 1H), 7.71 (s, 1H), 7.58 (d, *J* = 8.3 Hz, 2H), 7.30 (d, *J* = 8.2 Hz, 2H), 5.50 (s, 2H), 5.38 (s, 2H), 3.01 (t, *J* = 11.9 Hz, 2H), 2.63 (ddd, *J* = 11.4, 7.5, 3.4 Hz, 1H), 2.58–2.45 (m, 4H), 1.98 (d, *J* = 12.6 Hz, 2H), 1.64–1.50 (m, 6H), 1.41 (d, *J* = 5.1 Hz, 2H). ^13^C NMR (101 MHz, CDCl_3_) δ 153.74, 152.86, 150.88, 141.19, 137.72, 132.74 (2C), 127.99 (2C), 119.69, 118.28, 112.17, 62.82 (2C), 50.14 (2C), 46.43 (2C), 44.96, 28.04, 26.03 (2C), 24.53. HRMS calcd. for (C_23_H_27_N_7_ [M + H]^+^): 402.2401. Found: 402.2398. HPLC purity: 96.83%.

6-([1,4’-Bipiperidin]-1’-yl)-9-(2,6-dichlorobenzyl)-9*H*-purine **3d**. White solid, yield 59%, m.p. 172–173 °C. ^1^H NMR (400 MHz, CDCl_3_) δ 8.23 (s, 1H), 7.30 (s, 1H), 7.23 (s, 1H), 7.17–7.09 (m, 1H), 5.45 (s, 2H), 5.37 (s, 2H), 2.85 (t, *J* = 12.4 Hz, 2H), 2.51 (t, *J* = 11.4 Hz, 1H), 2.40 (s, 4H), 1.83 (d, *J* = 12.3 Hz, 2H), 1.54–1.40 (m, 7H), 1.28 (d, *J* = 4.4 Hz, 2H). ^13^C NMR (101 MHz, CDCl_3_) δ 153.71, 152.53, 150.93, 137.05, 136.85, 130.85, 130.82, 128.84 (2C), 119.52, 62.98 (2C), 50.11 (2C), 44.94, 42.47 (2C), 27.97, 26.01 (2C), 24.54. HRMS calcd. for (C_22_H_26_Cl_2_N_6_ [M + H]^+^): 445.1669. Found: 445.1668. HPLC purity: 95.68%.

6-([1,4’-Bipiperidin]-1’-yl)-2-chloro-9-(4-fluorobenzyl)-9*H*-purine **3e**. White solid, yield 58%, m.p. 140–142 °C. ^1^H NMR (400 MHz, CDCl_3_) δ 7.82 (s, 1H), 7.47 (dd, *J* = 8.3, 5.5 Hz, 2H), 7.23 (t, *J* = 8.5 Hz, 2H), 5.65 (bs, 2H), 5.47 (s, 2H), 3.24 (s, 2H), 2.90–2.81 (m, 1H), 2.75 (s, 4H), 2.20 (d, *J* = 12.4 Hz, 2H), 1.90–1.71 (m, 6H), 1.65 (d, *J* = 4.6 Hz, 2H). ^13^C NMR (101 MHz, CDCl_3_) δ 163.86–161.40 (d, ^1^*J_CF_* = 247.6 Hz), 154.19, 153.82, 152.14, 138.04, 131.35–131.32 (d, ^4^*J_CF_* = 3.3 Hz), 129.78–129.69 (d, ^3^*J_CF_* = 8.3 Hz), 118.54, 116.09–115.88 (d, ^2^*J_CF_* = 21.7 Hz), 62.63, 50.15, 46.39, 44.79, 28.04, 26.12, 24.59. ^19^F NMR (376 MHz, CDCl_3_) δ −113.25 (1F). HRMS calcd. for (C_22_H_26_ClFN_6_ [M + H]^+^): 429.1964. Found: 429.1963. HPLC purity: 98.26%.

6-([1,4’-Bipiperidin]-1’-yl)-2-chloro-9-(4-chlorobenzyl)-9*H*-purine **3f**. White solid, yield 59%, m.p. 139–141 °C. ^1^H NMR (400 MHz, CDCl_3_) δ 7.60 (s, 1H), 7.28 (d, *J* = 8.4 Hz, 2H), 7.18 (d, *J* = 8.4 Hz, 2H), 5.42 (s, 2H), 5.25 (s, 2H), 3.01 (s, 2H), 2.63 (ddd, *J* = 11.5, 8.3, 3.5 Hz, 1H), 2.57–2.46 (m, 4H), 1.97 (d, *J* = 12.6 Hz, 2H), 1.67–1.49 (m, 6H), 1.42 (d, *J* = 5.0 Hz, 2H). ^13^C NMR (101 MHz, CDCl_3_) δ 154.22, 153.81, 152.14, 138.04, 134.37, 134.00, 129.20 (2C), 129.19 (2C), 118.52, 62.62 (2C), 50.14 (2C), 46.38 (2C), 44.85, 28.03, 26.11 (2C), 24.58. HRMS calcd. for (C_22_H_26_Cl_2_N_6_ [M + H]^+^): 445.1669. Found: 445.1669. HPLC purity: 98.96%.

4-((6-([1,4’-Bipiperidin]-1’-yl)-2-chloro-9*H*-purin-9-yl)methyl)benzonitrile **3g**. White solid, yield 66%, m.p. 187–188 °C. ^1^H NMR (400 MHz, CDCl_3_) δ 7.69–7.53 (m, 3H), 7.32 (d, *J* = 7.2 Hz, 2H), 5.62 (bs, 2H), 5.36 (bs, 2H), 3.04 (bs, 2H), 2.66 (bs, 1H), 2.56 (bs, 4H), 2.01 (d, *J* = 11.2 Hz, 2H), 1.62 (bs, 6H), 1.44 (bs, 2H). ^13^C NMR (101 MHz, CDCl_3_) δ 154.43, 153.82, 152.16, 140.77, 137.92, 132.81 (2C), 128.17 (2C), 118.50, 118.23, 112.40, 62.62 (2C), 50.17 (2C), 46.46 (2C), 27.99, 26.00 (2C), 24.51. HRMS calcd. for (C_23_H_26_ClN_7_ [M + H]^+^): 436.2011. Found: 436.2008. HPLC purity: 96.58%.

6-([1,4’-Bipiperidin]-1’-yl)-2-chloro-9-(2,6-dichlorobenzyl)-9*H*-purine **3h**. White solid, yield 52%, m.p. 88–89 °C. ^1^H NMR (400 MHz, CDCl_3_) δ 7.22 (d, *J* = 11.4 Hz, 2H), 7.14 (dd, *J* = 8.8, 7.3 Hz, 1H), 5.39 (s, 2H), 5.21 (bs, 2H), 2.85 (s, 2H), 2.53–2.42 (m, 1H), 2.41–2.26 (m, 4H), 1.81 (d, *J* = 12.6 Hz, 2H), 1.52–1.34 (m, 7H), 1.27 (d, *J* = 5.0 Hz, 2H). ^13^C NMR (101 MHz, CDCl_3_) δ 154.01, 153.77, 152.15, 137.19, 136.88, 131.01, 130.35, 128.86 (2C), 118.38, 62.67 (2C), 50.12 (2C), 45.06, 42.74 (2C), 27.99, 26.10 (2C), 24.58. HRMS calcd. for (C_22_H_25_Cl_3_N_6_ [M + H]^+^): 479.1279. Found: 479.1280. HPLC purity: 96.93%.

#### 3.1.3. Human Histamine H_3_ Radioligand Displacement

Radioligand displacement assays at the human histamine H_3_R were performed, as published previously [28]. In summary, the compounds were incubated with membrane preparations of HEK-293 cells stably expressing the human histamine H_3_R (20 μg/well) and [^3^H]*N*^α^-methylhistamine (2 nM, K_D_ = 3.08 nM) in 200 μL of binding buffer (10 mM MgCl_2_, 100 mM NaCl and 75 mM Tris/HCl, pH 7.4) for 90 min. At least three independent experiments of each compound in duplicate were conducted with 11 concentrations. Obtained data was analyzed with GraphPad Prism 7 (San Diego, CA, USA) using non-linear least squares fit and the equation “one site competition” (representative Figures given in the SI). K_i_ values were calculated according to the Cheng–Prusoff equation [29] and are reported as means with a 95% confidence interval. Statistical analyses were performed on the -logK_i_ values and were converted to mean K_i_ values and a 95% confidence interval. Differences are considered significant if 95% confidence intervals are not overlapping.

#### 3.1.4. Cell Cultures

Cultures of HEPG2 (ATCC-HB-8065 TM), HEK293 (ATCC CRL-1573 TM) and SHSY-5Y (ATCC CRL-2266 TM) cell lines were used as experimental models. Cells were maintained in a DMEM culture medium supplemented with 10% Foetal Bovine Serum (FBS), in an incubator at 37 °C and a humidified atmosphere with 5% CO_2_. Cells were seeded on culture plates, were allowed to grow for 24 h and then were treated with the synthesized compounds for 48 h with 100, 10, 1 and 0.1 µM concentrations. Negative cell death control, corresponding to untreated cells, and positive cell death control, corresponding to cells treated with 10% SDS, were maintained.

#### 3.1.5. Cytotoxicity Assays

In a colorimetric MTT reduction assay [30], sterile 96-well culture plates were used. An amount of 2000–2500 cells per well were seeded. Upon completion of treatment, the culture medium was vacuum aspirated and washed with 1x PBS, and then 100 μL of 0.5 mg/mL MTT solution was added for a period of 4 h at 37 °C for formazan crystal formation. At the end of the 4 h, 100 μL of 10% SDS in HCl was added and kept overnight in an incubator at 37 °C to dissolve the formed crystals. The absorbance of each well was measured on the Synergy HTX microplate reader at 570 nm wavelength.

#### 3.1.6. Docking Studies

The 3D structures of the compounds that were synthesized were built using OECHEM, followed by protonation states, and they were adjusted to a pH of 7.2 using FixpKa from the QUACPAC package (QUACPAC 2.1.3.0: OpenEye Scientific Software, Santa Fe, NM. http://www.eyesopen.com, accessed on 1 November 2021). The conformers were generated using OMEGA software [31]. The H_3_R model was downloaded from the GPCR database (https://gpcrdb.org, accessed on 1 May 2022) [32,33,34]. The preparation of the proteins was performed with Schrodinger Suite 2021–1 [35], and the minimization was performed in the presence of restraints to maintain the protein conformation utilizing force field, OPLS4 [36].

The molecular docking studies were carried out with the induced-fit docking protocol [37,38], considering possible interactions with Asp114 or Glu206. The best poses were refined with the MM-GBSA method [39] in order to obtain the binding free energy by taking into account the solvation energies of the interacting molecules in addition to the molecular mechanics (MM) energies. The contribution of polar solvation energies was computed by the generalized Born (GB) implicit solvent model, whereas the nonpolar contribution of the solvation energy was dependent on solvent-accessible surface area (SA). The interactions of residues in the active site were identified using a 6 Å radius around the docked position as a reference.

#### 3.1.7. Molecular Dynamics

The molecular dynamic simulation was carried out with Desmond software (Schrödinger Release 2021-1: Desmond Molecular Dynamics System, D. E. Shaw Research, New York, NY, USA, 2021. Maestro-Desmond Interoperability Tools, Schrödinger, New York, NY, USA, 2021). The ligand–receptor complexes were inserted into the POPC membrane, were solvated and were ionized up to a concentration of 0.15 M NaCl using the Membrane Builder module from the System Builder. The protein orientation was defined based on the OPM database [40]. Orthorhombic periodic boundary conditions were set up to specify the shape and size of the repeating unit buffered at 10 Å distances. The simulated systems were first relaxed with 2000 steps of minimization, and then they were gradually heated from 0 to 310 K by repeating 500-step MD simulations every cycle. Except for the lipid tails, which were left to equilibrate for 250 ps, the systems were then fixed. The simulation was then changed to NPT conditions and equilibrated for 2.5 ns while constraining the protein with an initial force constant of 10 kcal/mol^2^ that was gradually reduced to 8, 6, 4, 2, 1, 0.5 and 0.05 kcal/mol^2^ every 250 ps of the MD simulation. Finally, the systems were run for 50 ns without any limits. The OLPS4 force field described proteins, lipids, and ions. In the production phase, a timestep of 2 fs was used, and PME (Particle Mesh Ewald) was used to tackle long-range electrostatic interactions. Langevin dynamics were used to keep the temperature at 310 K. The Simulation Interaction Diagram module (Schrodinger Suite 2021-1) and the PyMOL program (The PyMOL Molecular Graphics System, Version 2.0 Schrödinger, LLC., New York, NY, USA) were used to analyze the results.

## 4. Conclusions

In this work, we synthesized eight new purine derivatives in a simple manner and in good yields that include the classical pharmacophore of the H_3_R ligands. The results of the affinity assays show that all these derivatives bind to receptors with K_i_ values on the nanomolar scale (2.91–105 nM), and compound **3d** presented the best affinity with a K_i_ value of 2.91 nM, which is better than pitolisant. Furthermore, **3d** showed low cytotoxicity for the kidney, liver and neuroblastoma cell lines. By in silico studies, the importance of the N-7 purine scaffold in the stabilization of the ligand–receptor complex through a hydrogen bond interaction with the Tyr374 residue at the binding site could be determined. Finally, the predicted ADME properties indicated that these derivatives fulfill all the requirements for good absorption and penetration into the CNS.

## Figures and Tables

**Table 1:** Affinities to the H_3_R of compounds **3a-h** and reference ligand pitolisant.

Compound	Substitution Pattern		K_i_ (nM) ^a^ [95% CI nM]
	X	R	
3a	H	4-F	105 [79.6–140]
3b	H	4-Cl	39.4 [8.78–177]
3c	H	4-CN	28.8 [9.90–83.7]
3d	H	2,6-diCl	2.91[1.31–6.44]
3e	Cl	4-F	94.6 [47.5–189]
3f	Cl	4-Cl	44.6 [14.2–141]
3g	Cl	4-CN	77.8 [29.2–207]
3h	Cl	2,6-diCl	5.51 [1.17–25.9]
Pitolisant			6.09 [2.28–16.3]

^a^ K_i_ values were determined by [^3^H]*N*^α^-methylhistamine binding assay at the human histamine H_3_R. Values represent affinity as mean values and as corresponding confidence intervals (CI 95%) of at least three independent experiments in duplicate.

**Table 2:** Cytotoxicity of selected compounds **3d** and **3h**.

Cell line	IC_50_ (µM) ^a^	
	3d	3h
HEK-293	69.14 ± 13.95	54.31 ± 3.54
SH-SY5Y	>100	32.38 ± 4.75
HepG2	57.57 ± 12.02	33.76 ± 4.29

^a^ IC_50_ values were determined in three independent experiments in triplicate in the range 0.1 to 100 μM.

**Table 3:** Biological activity in K_i_ values and binding free energy calculated with MM-GBSA.

	Biological Activity		Binding Free Energy
	K_i_ (nM)	−logK_i_	kcal/mol
3a	105.0	6.98	−59.21
3b	39.4	7.40	−57.72
3c	28.8	7.54	−64.10
3d	2.9	8.54	−76.98
3e	94.5	7.02	−66.08
3f	44.6	7.35	−64.97
3g	77.8	7.11	−64.56
3h	5.5	8.26	−75.10

**Table 4:** In silico ADME and drug-likeness properties of compounds **3a–h**.

Compound	MW (g/mol)	NRB	HBA	HBD	TPSA	cLogP	GI Absorption	BBB Permeant
**3a**	394.49	4	5	0	50.08	3.26	High	Yes
**3b**	410.94	4	4	0	50.08	3.48	High	Yes
**3c**	401.51	4	5	0	73.87	2.76	High	Yes
**3d**	445.39	4	4	0	50.08	3.91	High	Yes
**3e**	428.93	4	5	0	50.08	3.82	High	Yes
**3f**	445.39	4	4	0	50.08	4.04	High	Yes
**3g**	435.95	4	5	0	73.87	3.30	High	Yes
**3h**	479.83	4	4	0	50.08	4.58	High	Yes

MW: molecular weight; NRB: number of rotatable bonds; HBA: number of hydrogen bond acceptors; HBD: number of hydrogen bond donors; cLogP: consensus Log *p* value; TPSA: topological polar surface.

## Data Availability

Data is contained within the article and supplementary material.

## Supplementary Materials

The following supporting information can be downloaded at: https://www.mdpi.com/article/10.3390/ph15050573/s1, Figure S1: Graphical representation of histamine at the H3R binding site; Figure S2: The two best poses were achieved with the induced-fit docking protocol for 3d at the H3R binding site. (A) Piperidine oriented toward Asp114, and (B) piperidine oriented toward Glu206; Figure S3: The RMSD of the two studied complexes (3a in red and 3d in blue) was obtained during 50 ns of MD simulations; Figure S4: The root means square deviation (RMSF) values of each residue averaged over two trajectories for 3a (red line) and 3d (blue line); Figure S5: (A) Histogram plot of the contact H3R-3a system. (B) Timeline plots represent of the interactions and contact with 3a; Figure S6: (A) Histogram plot of the contact H3R-3d system. (B) Timeline plots represent of the interactions and contact with 3d; Figure S7: Graphical representations of 3a and 3d properties in the binding site.
