# Supplementary material for: Substituted Purines as High-Affinity Histamine H3 Receptor Ligands
Source: Pharmaceuticals (Basel). 2022 May 4;15(5):573. doi: 10.3390/ph15050573 (PMC9145483; doi:10.3390/ph15050573)
Supplement: Supplementary file 1 [file pharmaceuticals-15-00573-s001.zip › pharmaceuticals-1695547-supplementary.pdf]

Supporting information

Substituted purines as high affinity histamine H_3 receptor ligands

Christian Espinosa-Bustos,[1*] Luisa Leitzbach,[2] Tito Añazco,[1] María J. Silva,[1] Andrea del Campo,[1] Alejandro Castro-Alvarez,[3] Holger Stark,[2] Cristian O. Salas [4*]

[1] Departamento de Farmacia, Facultad de Química y de Farmacia, Pontificia Universidad Católica de Chile, Santiago de Chile 702843, Chile.; ccespino@uc.cl (C.E-B.), taanazco@uc.cl (T.A.); mjsilva15@uc.cl (M.J.S.); andrea.delcampo@uc.cl (A. del C.)

[2] Institute of Pharmaceutical and Medicinal Chemistry, Heinrich Heine University Düsseldorf, Universitaetsstr. 1, 40225 Duesseldorf, Germany.; luisa.leitzbach@hhu.de (L.L.); stark@hhu.de (H.S.)

[3] Laboratorio de Bioproductos Farmacéuticos y Cosméticos, Centro de Excelencia en Medicina Traslacional, Facultad de Medicina, Universidad de La Frontera, Temuco, Chile.; qf.alec.astro@gmail.com (A.C-A.)

[4] Departamento de Química Orgánica, Facultad de Química y de Farmacia, Pontificia Universidad Católica de Chile, 702843 Santiago de Chile, Chile.; cosalas@uc.cl (C.O.S.)

Index

^{1}H and ^{13}C NMR of compounds **3a-h**……………………………………………pag 02-09

HRMS of compounds **3a-h**………………………………………………………pag 10-13

HPLC of compounds **3a-h**………………………………………………………pag 14-17

In silico studies……………………………………………………………………pag 18-25

^{1}H NMR compound 3a

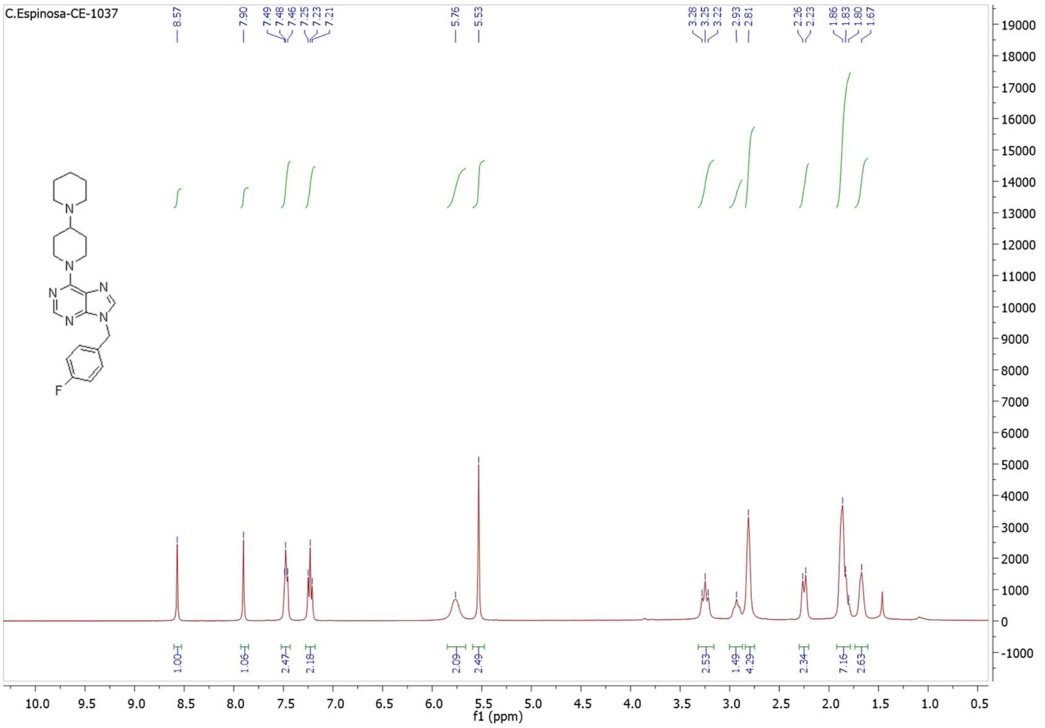

^{13}C NMR compound 3a

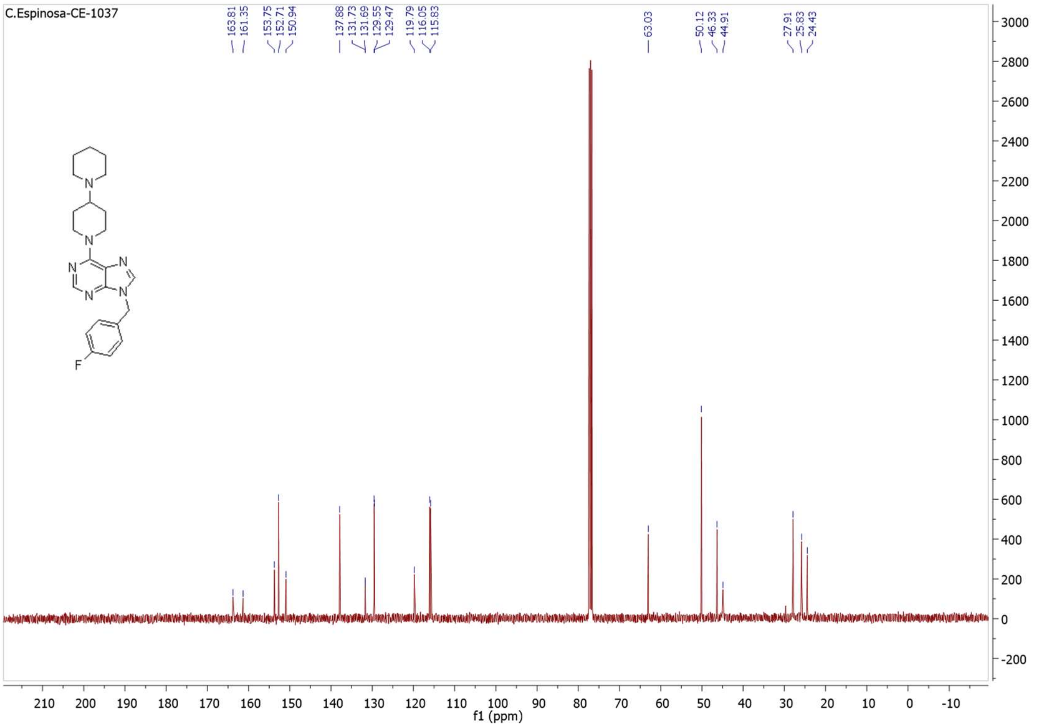

^{1}H NMR compound 3b

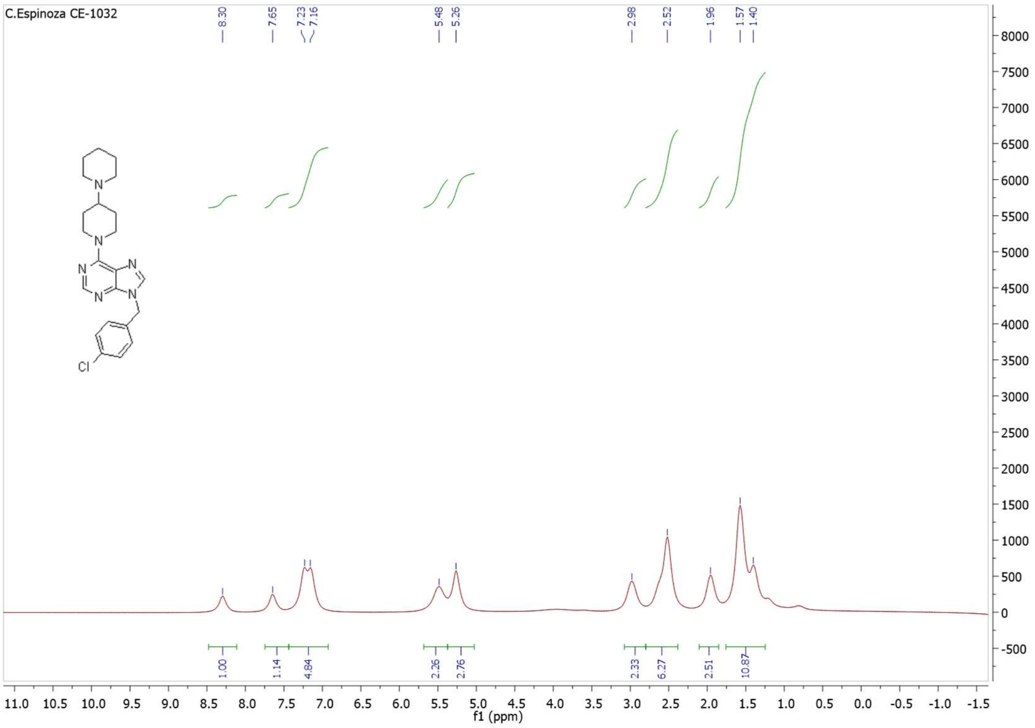

^{13}C NMR compound 3b

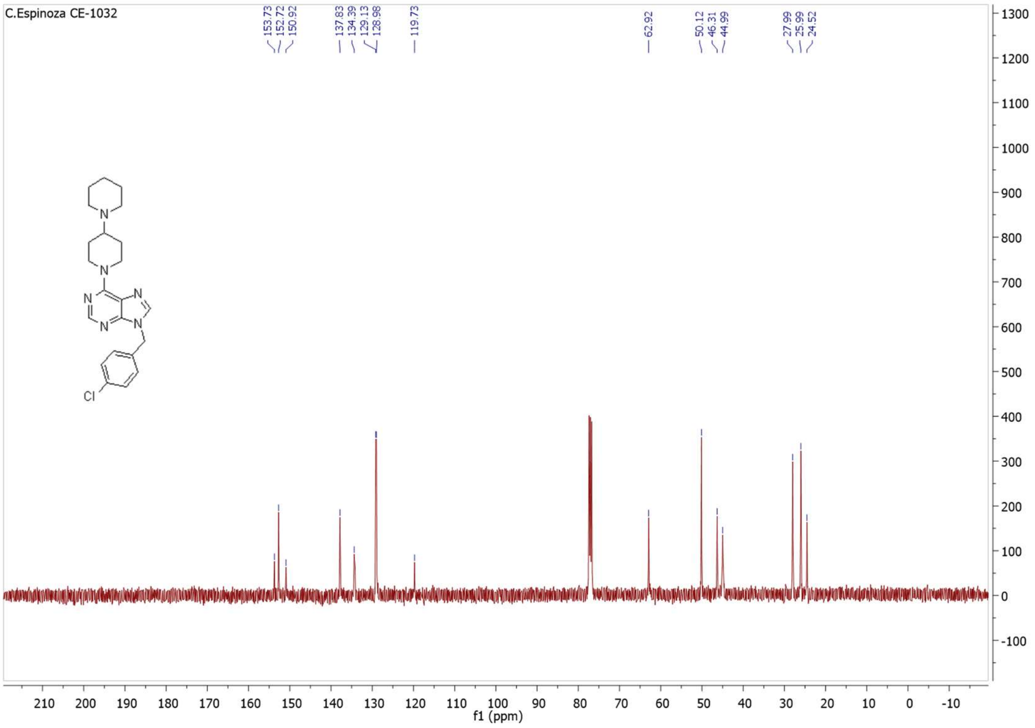

^{1}H NMR compound 3c

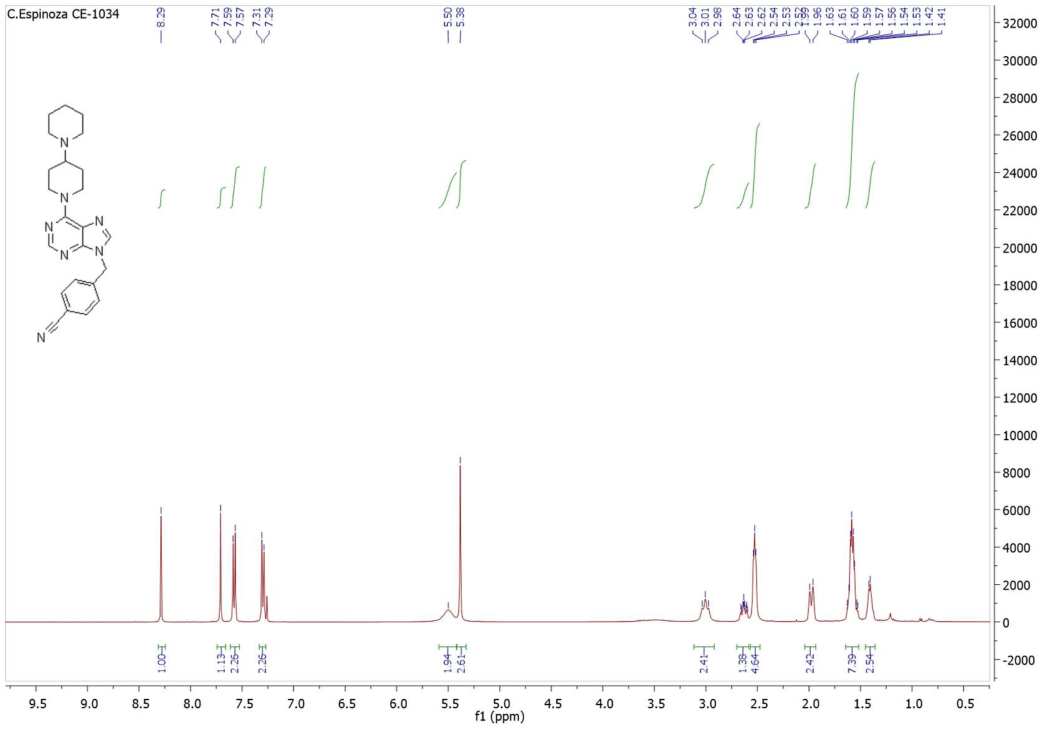

^{13}C NMR compound 3c

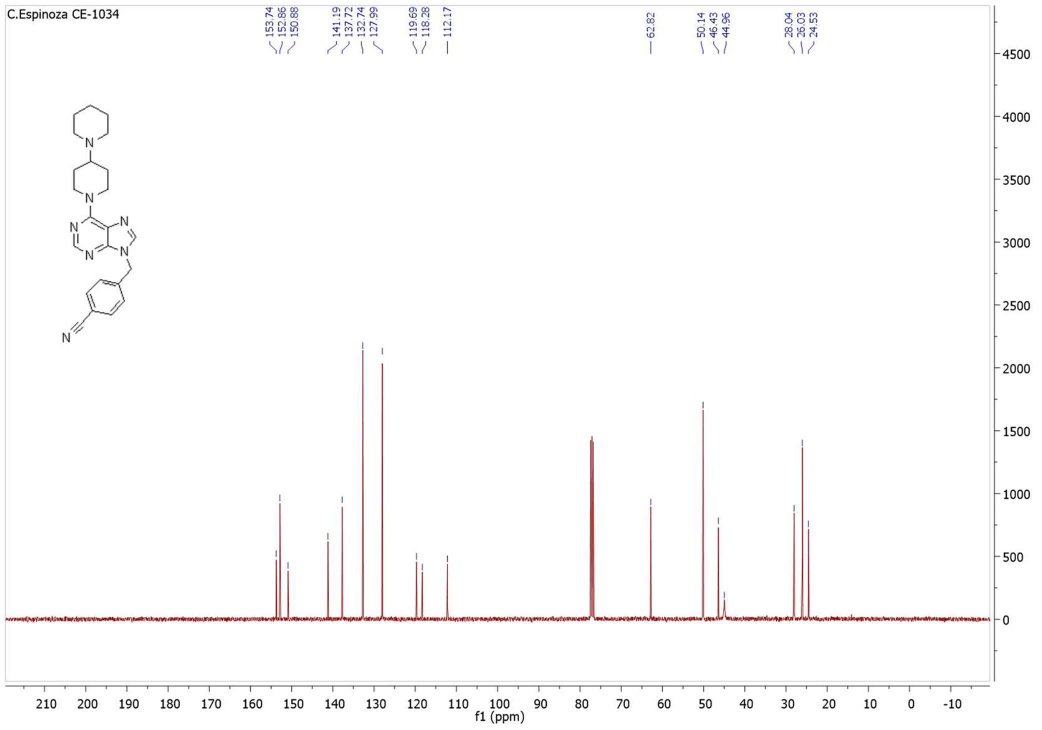

^{1}H NMR compound 3d

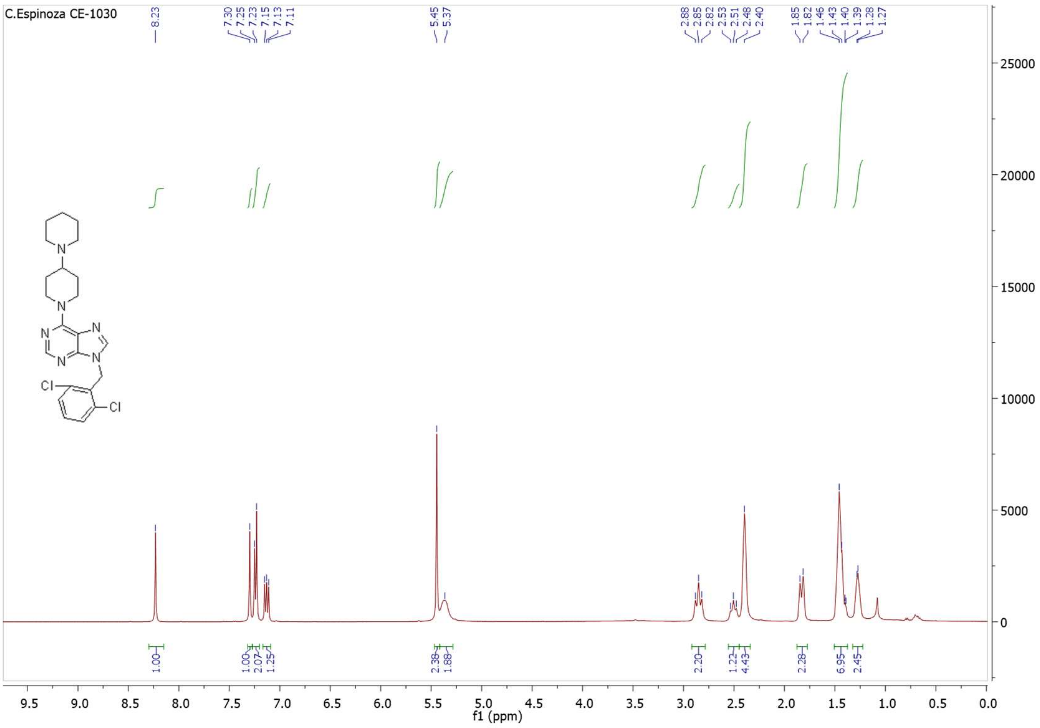

^{13}C NMR compound 3d

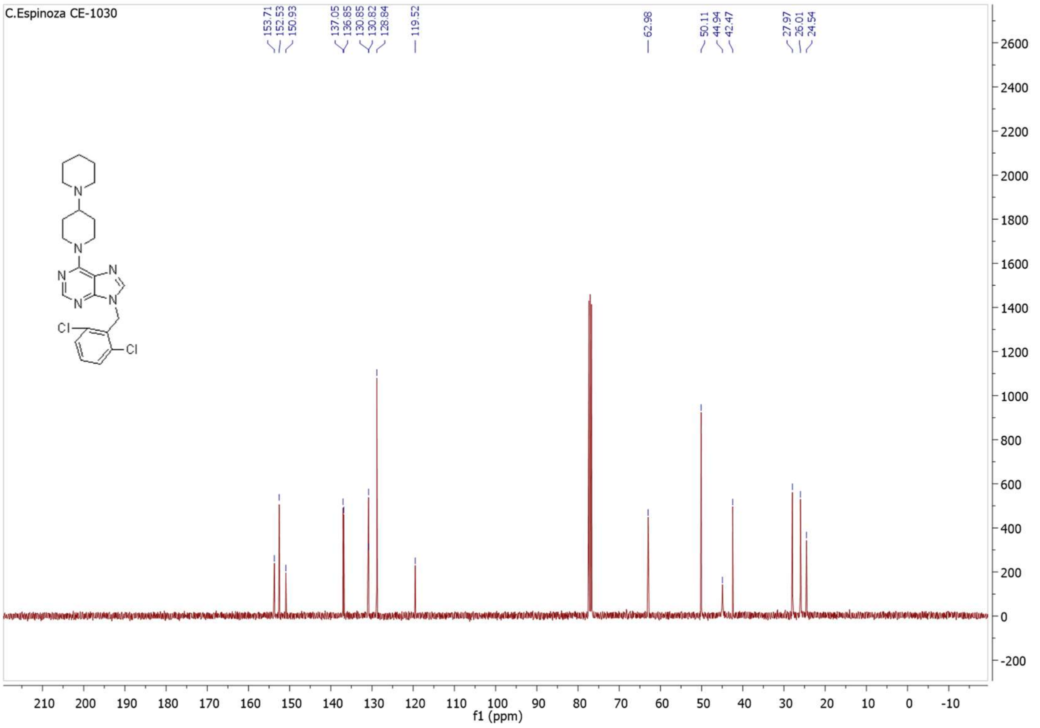

^{1}H NMR compound 3e

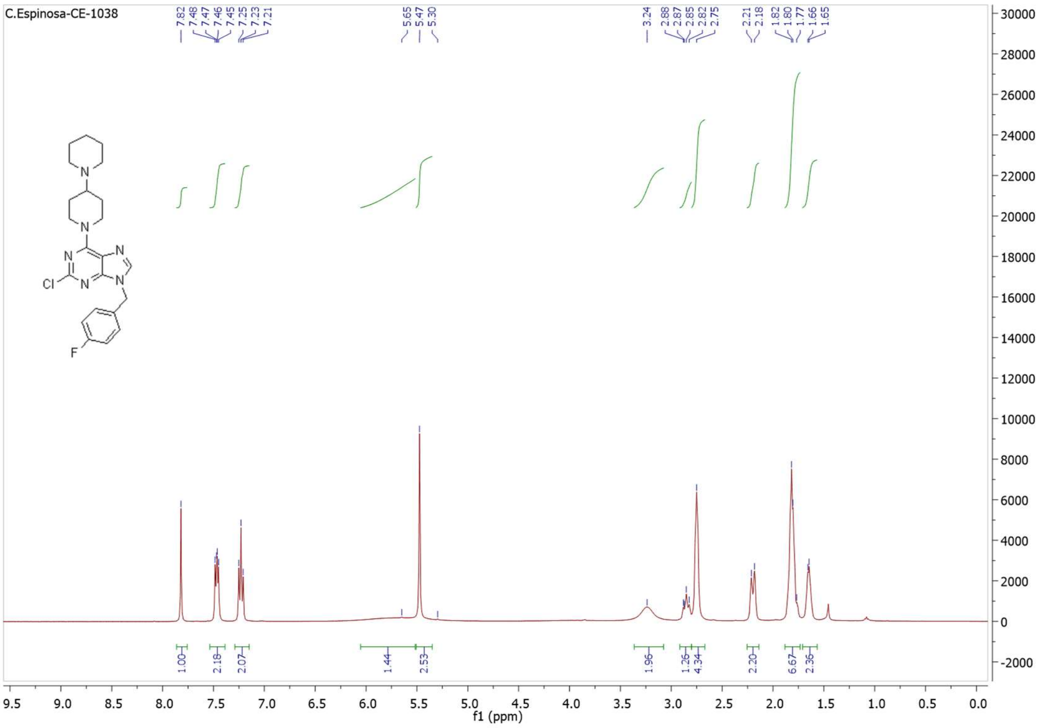

^{13}C NMR compound 3e

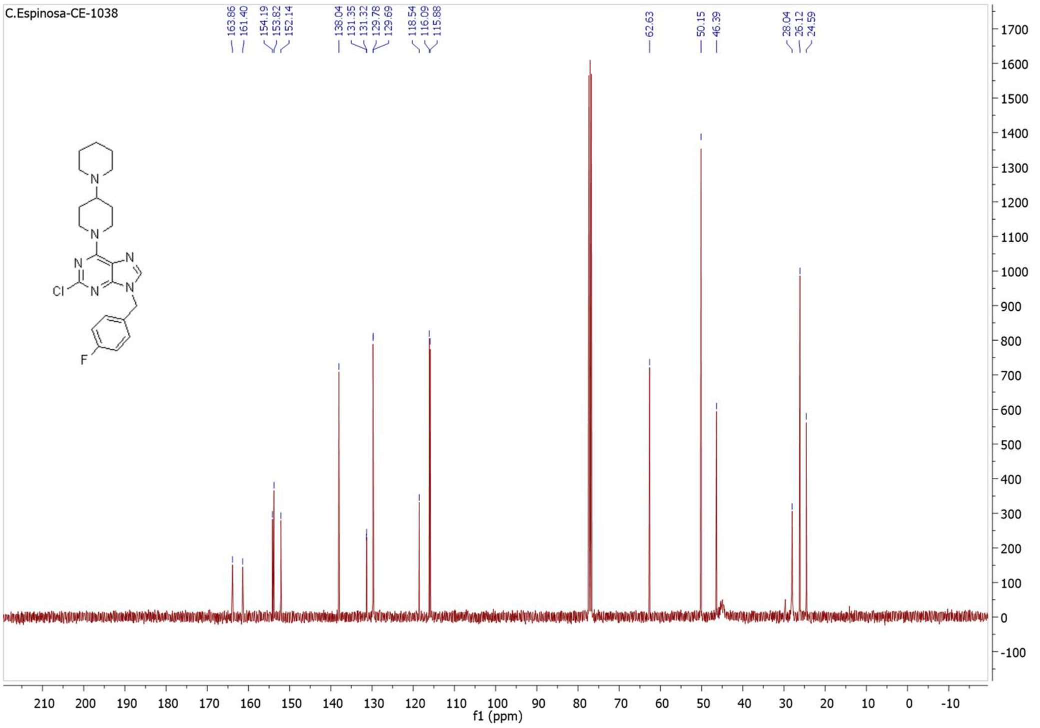

^{1}H NMR compound 3f

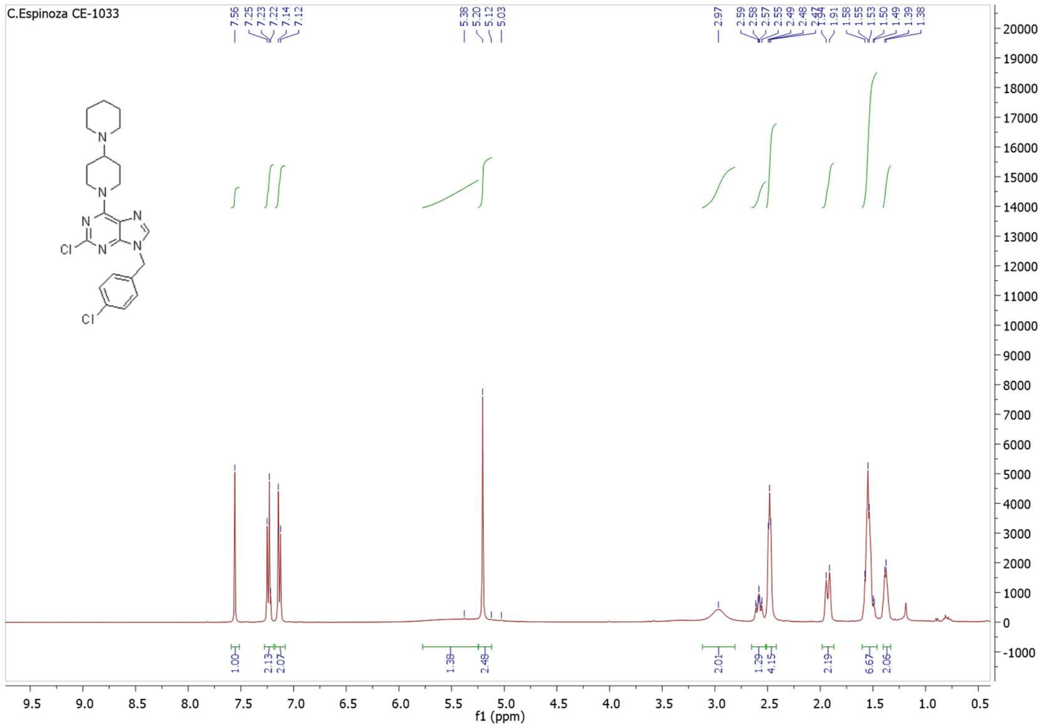

^{13}C NMR compound 3f

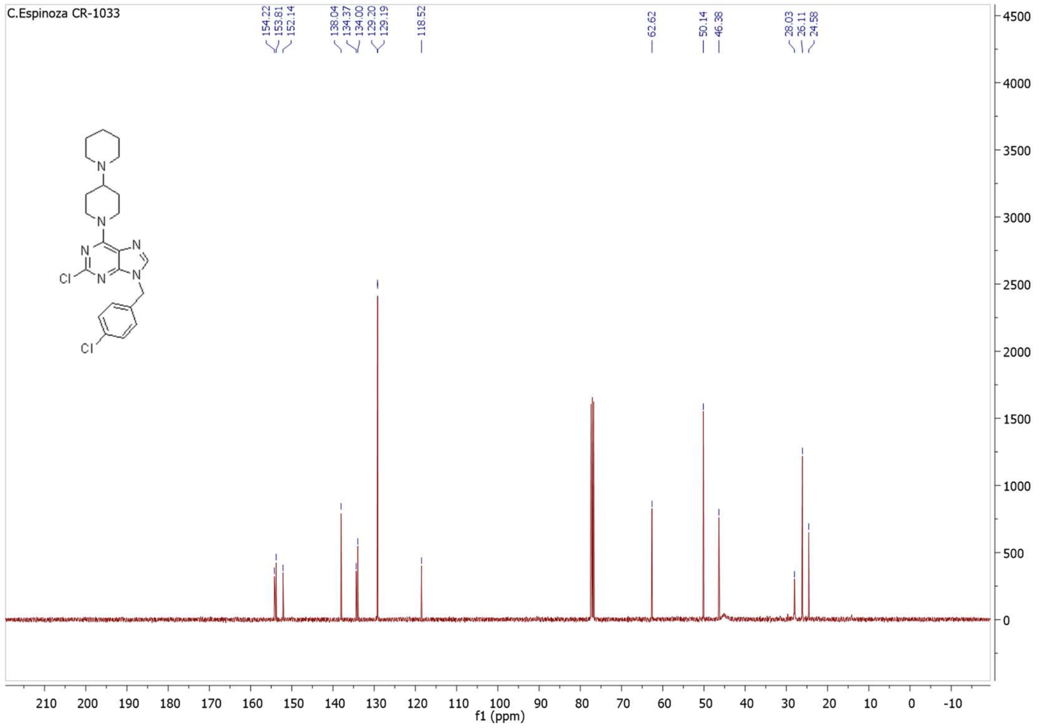

^{1}H NMR compound 3g

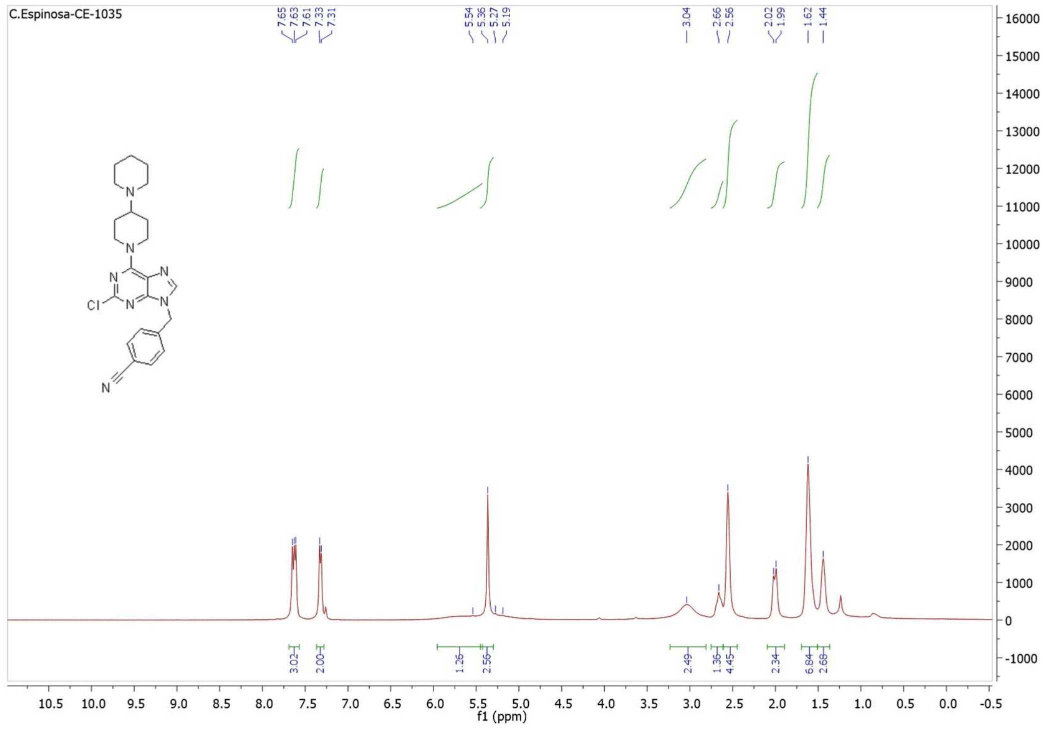

^{13}C NMR compound 3g

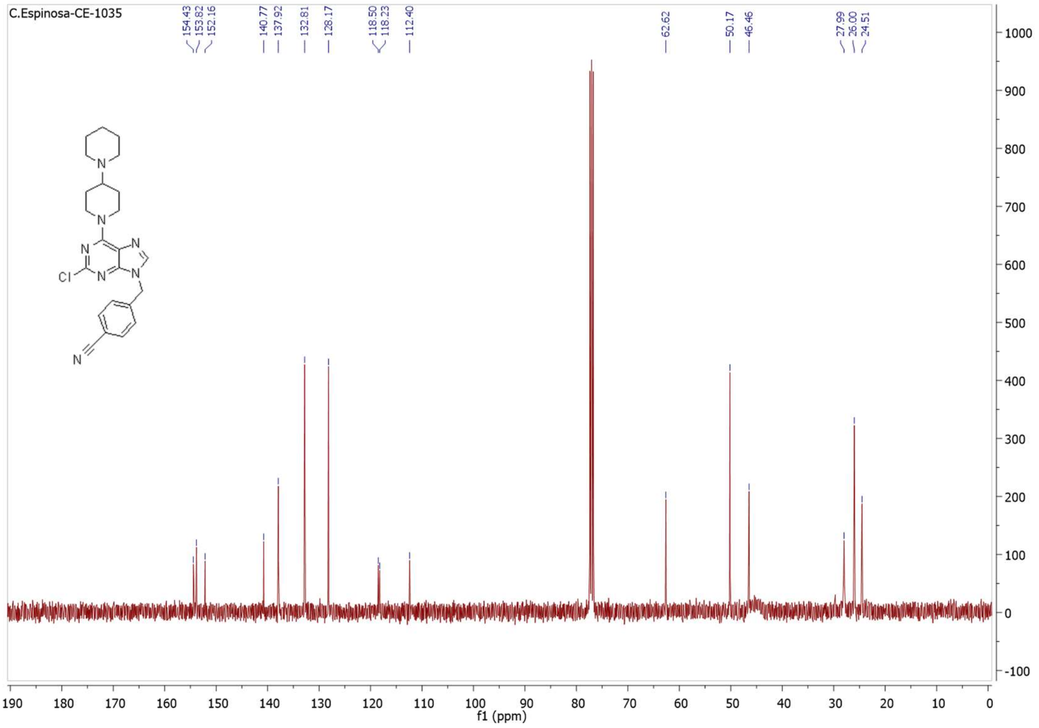

^{1}H NMR compound 3h

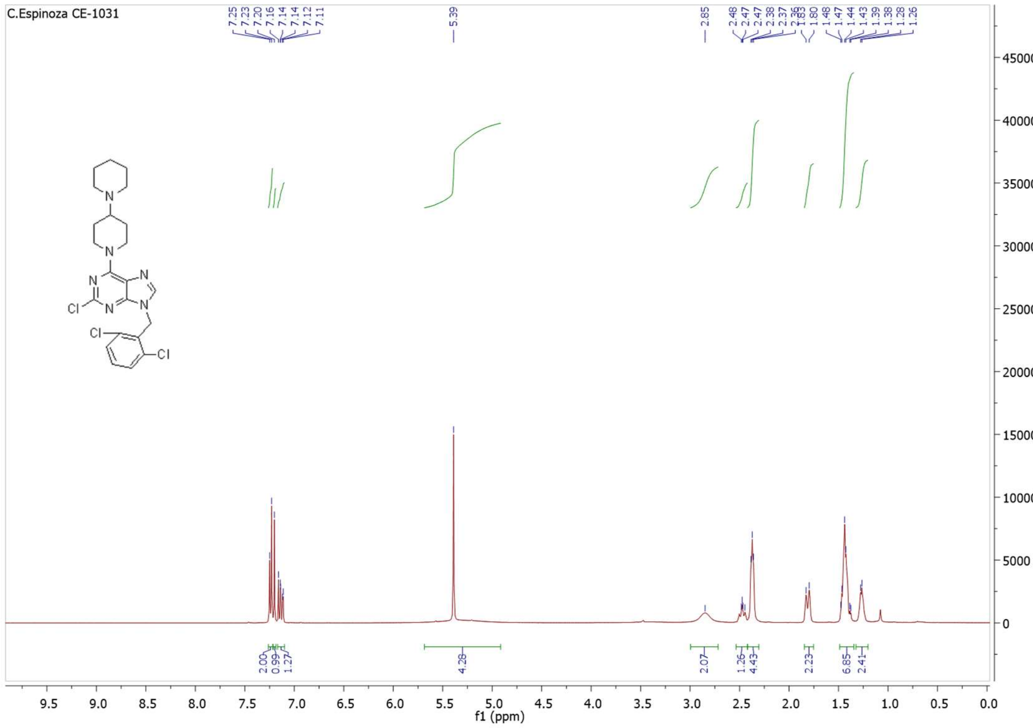

^{13}C NMR compound 3h

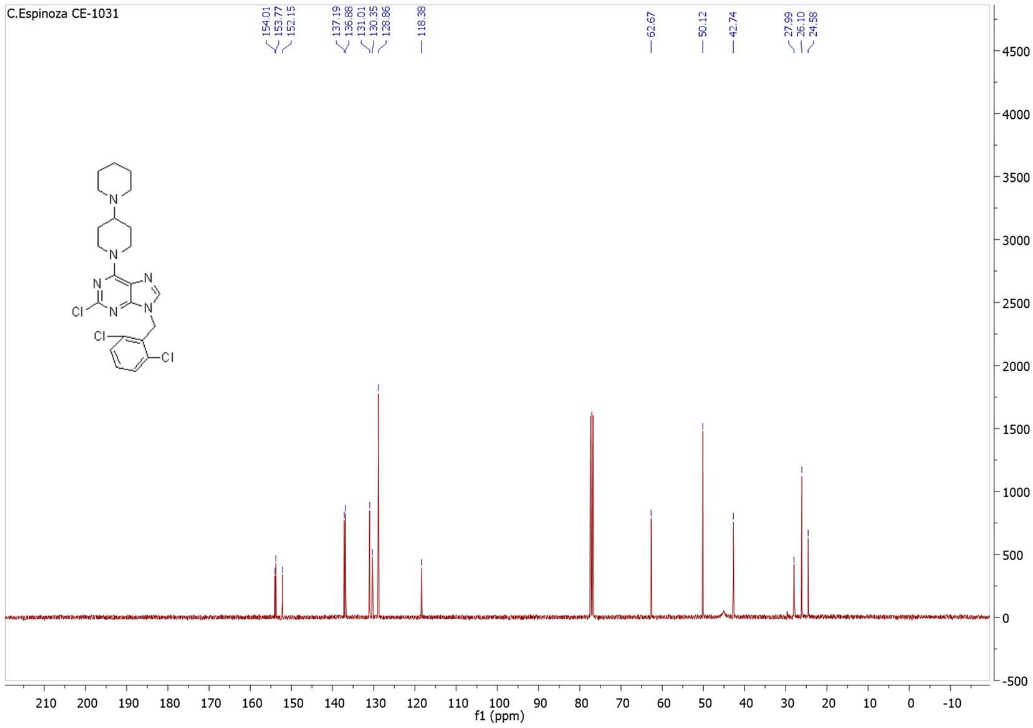

Mass spectra compound 3a

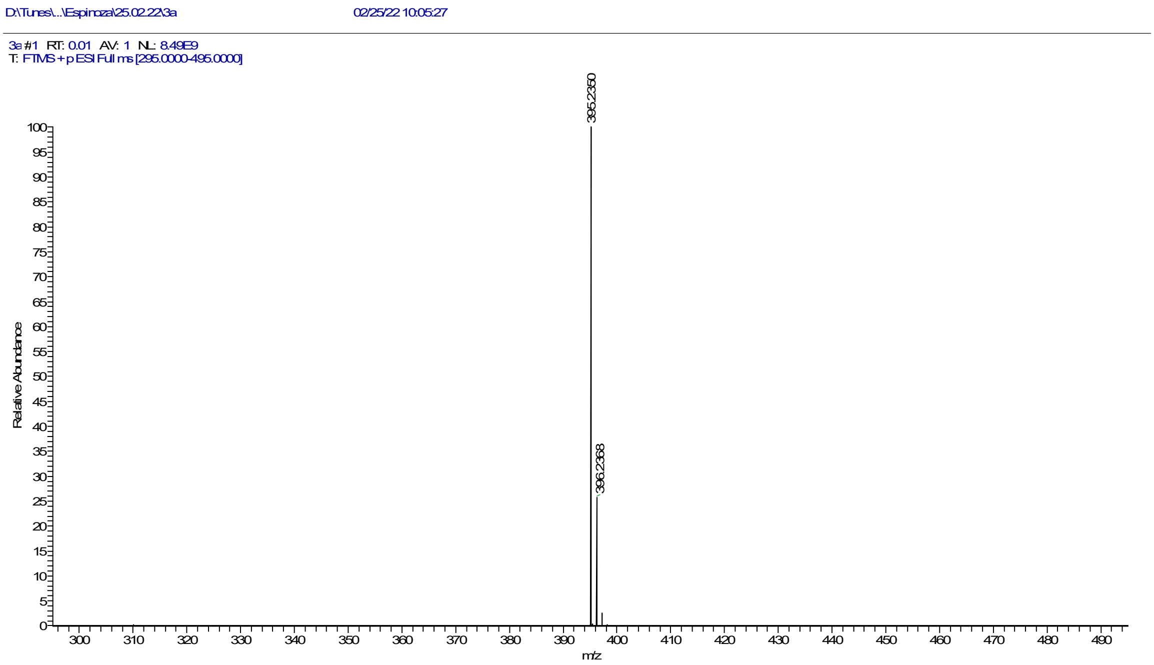

Mass spectra compound 3b

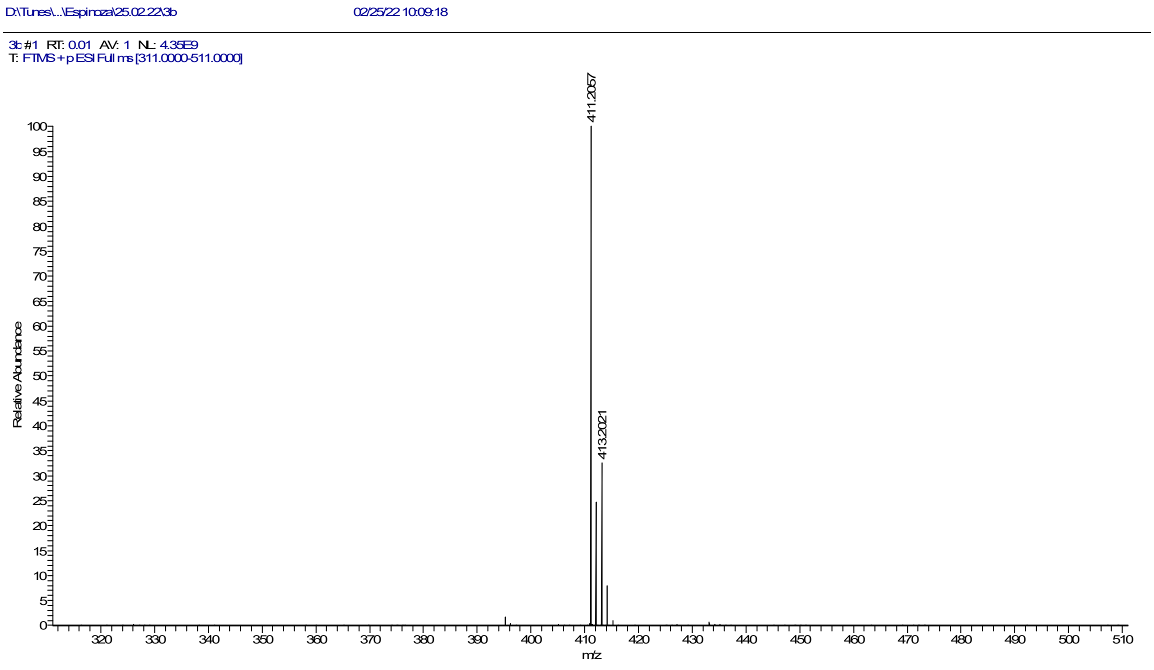

Mass spectra compound 3c

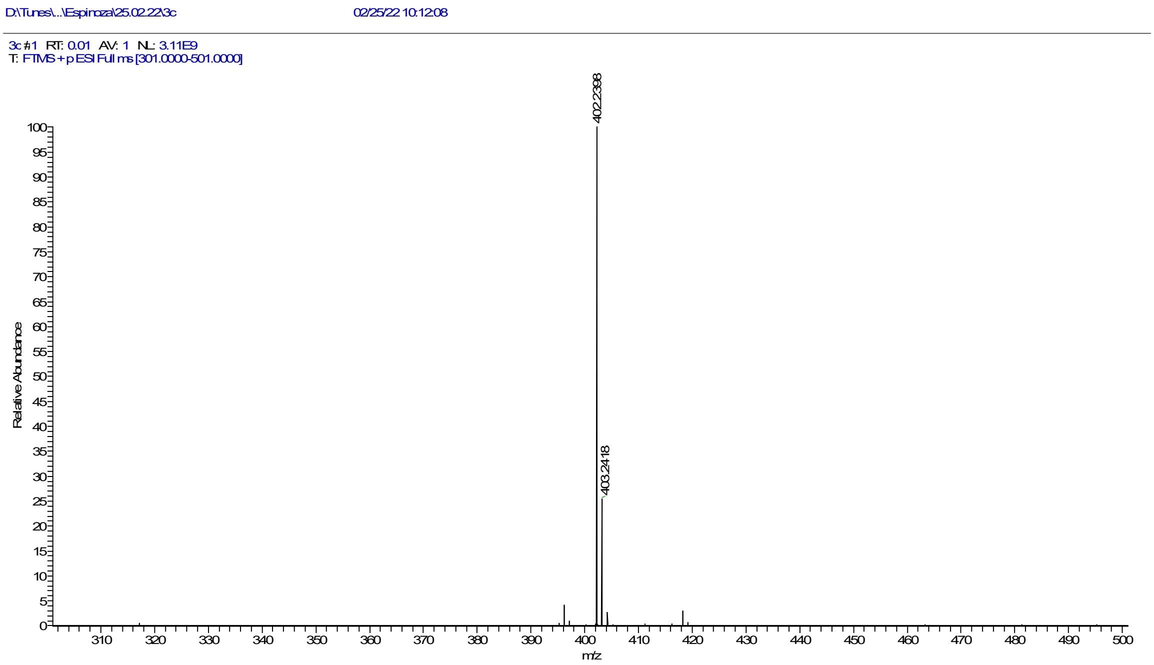

Mass spectra compound 3d

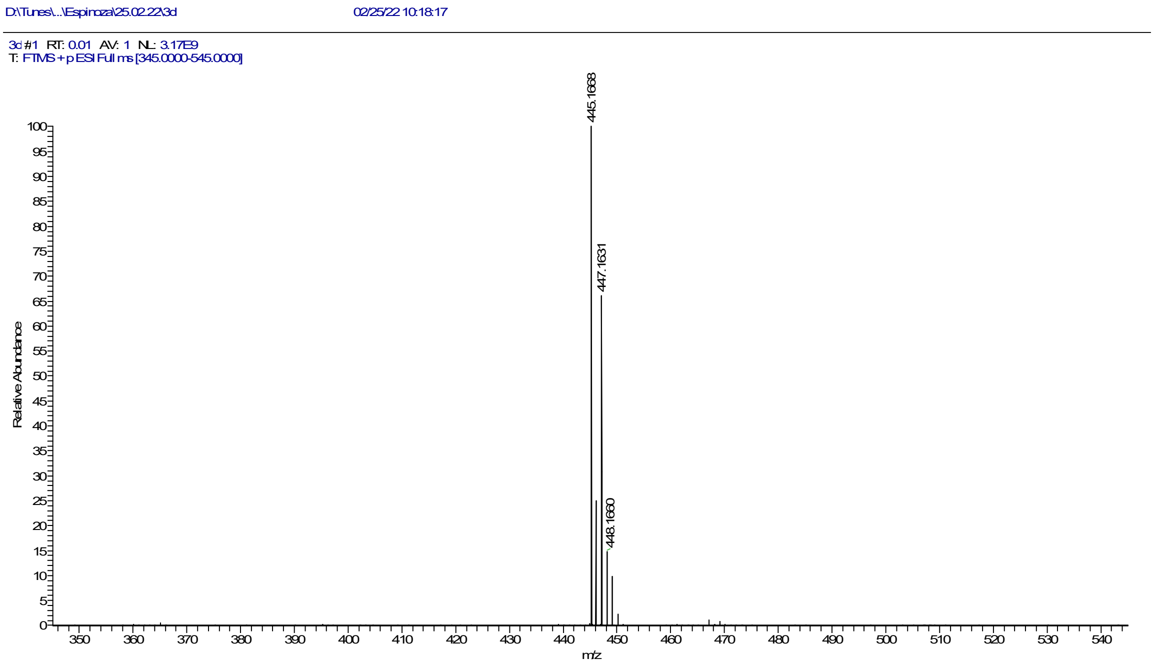

Mass spectra compound 3e

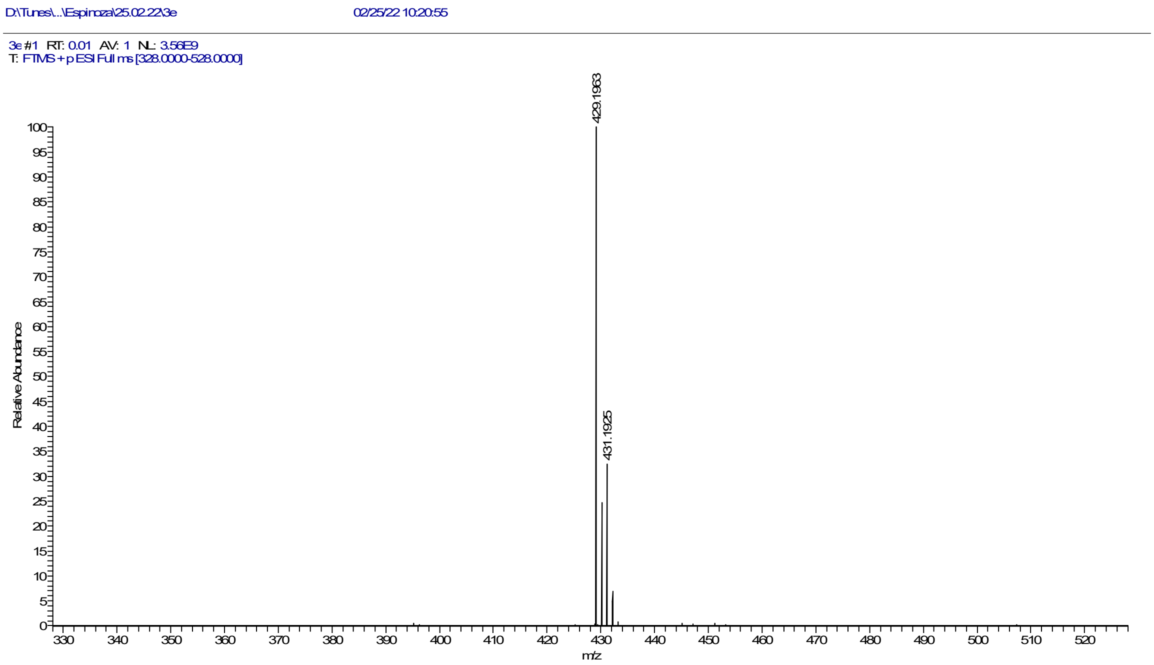

Mass spectra compound 3f

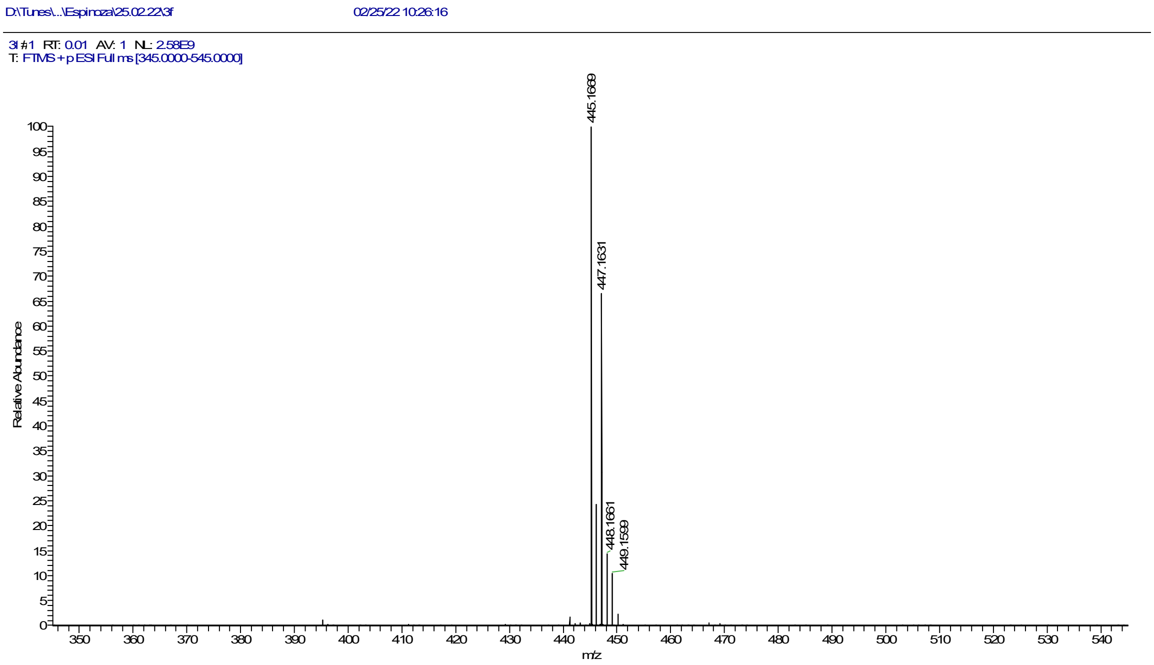

Mass spectra compound 3g

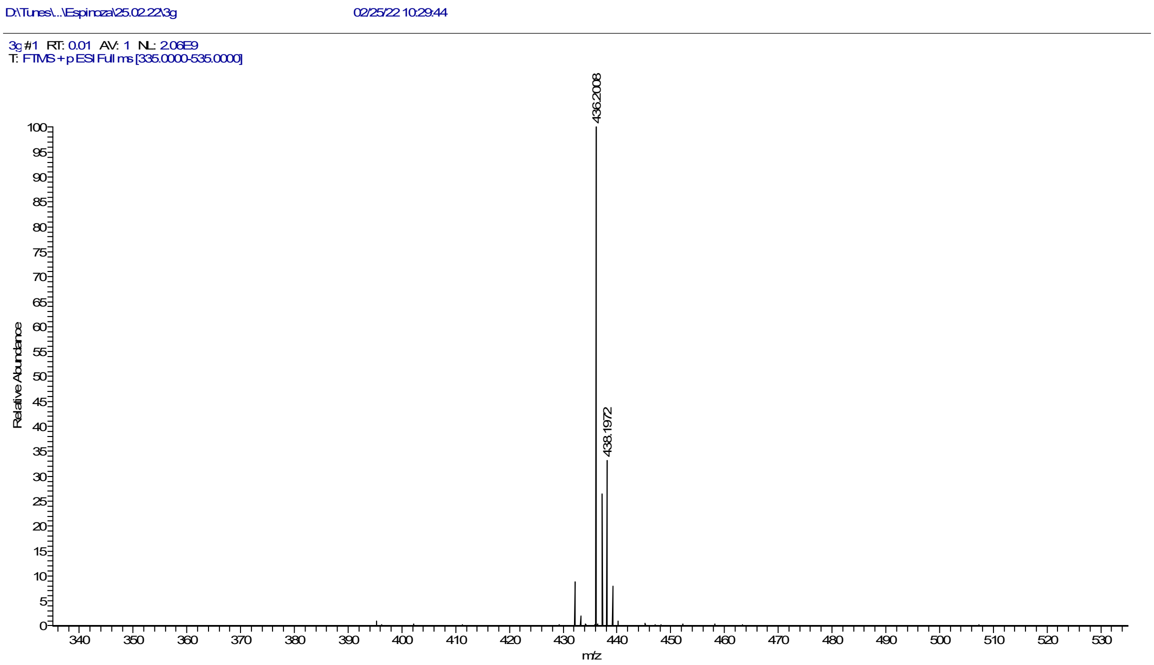

Mass spectra compound 3h

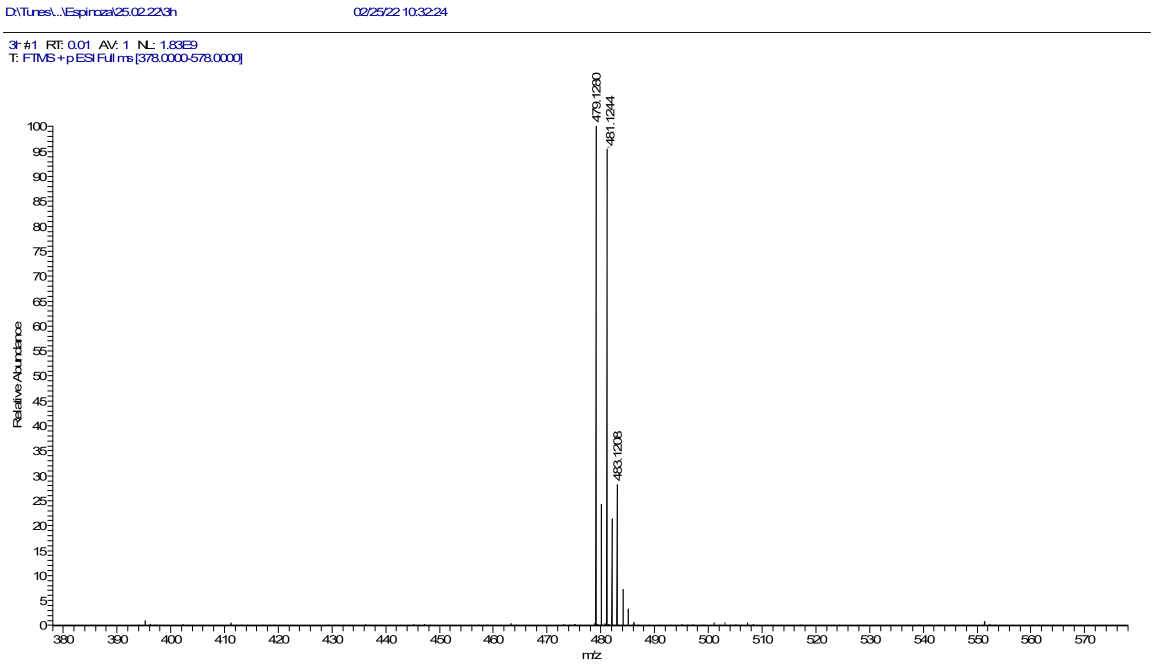

HPLC compound 3a

Shimadzu CLASS-VP V6.14 SP2 *Area % Report*

Page 1 of 1

Method Name: C:\CLASS-VP\Methods\Polyphenols_MA_02.met
Data Name: C:\CLASS-VP\Data\Joaquin_Fernandez\Tito\A001
User: Mario
Acquired: 26/04/2022 15:28:18
Printed: 27/04/2022 18:01:27

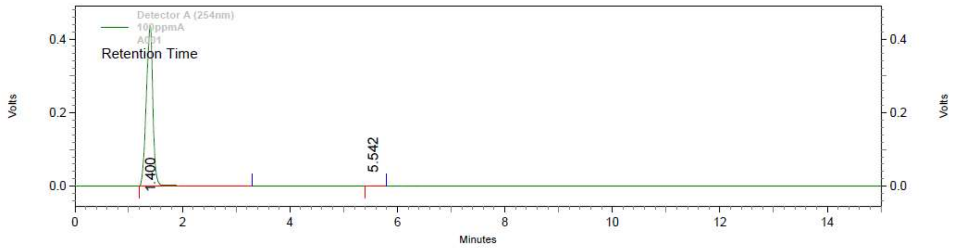

Detector A (254nm)

Pk #	Retention Time	Area	Area %	Height	Height %
1	1.400	3728760	99.979	436126	99.983
2	5.542	766	0.021	74	0.017
Totals		3729526	100.000	436200	100.000

HPLC compound 3b

Shimadzu CLASS-VP V6.14 SP2 *Area % Report*

Page 1 of 1

Method Name: C:\CLASS-VP\Methods\Polyphenols_MA_02.met
Data Name: C:\CLASS-VP\Data\Joaquin_Fernandez\Tito\B001
User: Mario
Acquired: 26/04/2022 15:49:21
Printed: 27/04/2022 18:05:27

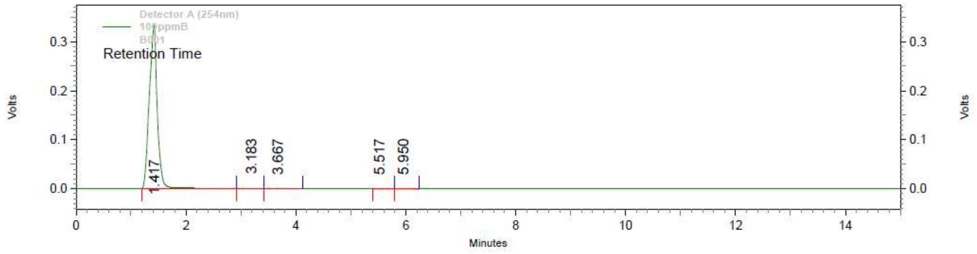

Detector A (254nm)

Pk #	Retention Time	Area	Area %	Height	Height %
1	1.417	3391621	99.397	333696	99.721
2	3.183	7493	0.220	382	0.114
3	3.667	3801	0.111	142	0.042
4	5.517	2954	0.087	116	0.035
5	5.950	6340	0.186	292	0.087
Totals		3412209	100.000	334628	100.000

HPLC compound 3c

Shimadzu CLASS-VP V6.14 SP2 *Area % Report*

Page 1 of 1

Method Name: C:\CLASS-VP\Methods\Polyphenols_MA_02.met
Data Name: C:\CLASS-VP\Data\Joaquin_Fernandez\Tito\C001
User: Mario
Acquired: 26/04/2022 16:11:04
Printed: 27/04/2022 18:06:37

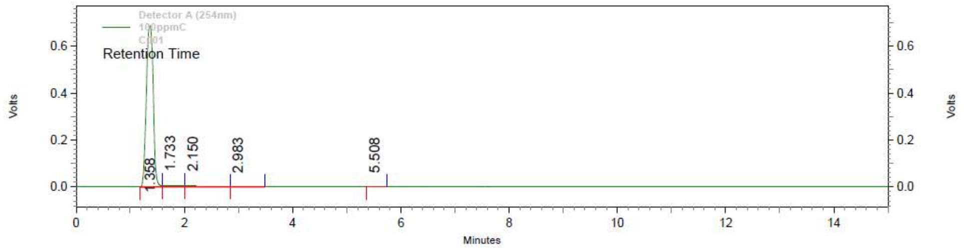

Detector A (254nm)

Pk #	Retention Time	Area	Area %	Height	Height %
1	1.358	6060354	96.830	687561	98.342
2	1.733	107465	1.717	6139	0.878
3	2.150	81529	1.303	4891	0.700
4	2.983	8742	0.140	489	0.070
5	5.508	669	0.011	72	0.010
Totals		6258759	100.000	699152	100.000

HPLC compound 3d

Shimadzu CLASS-VP V6.14 SP2 *Area % Report*

Page 1 of 1

Method Name: C:\CLASS-VP\Methods\Polyphenols_MA_02.met
Data Name: C:\CLASS-VP\Data\Joaquin_Fernandez\Tito\10D5050
User: Mario
Acquired: 27/04/2022 18:30:54
Printed: 27/04/2022 18:46:10

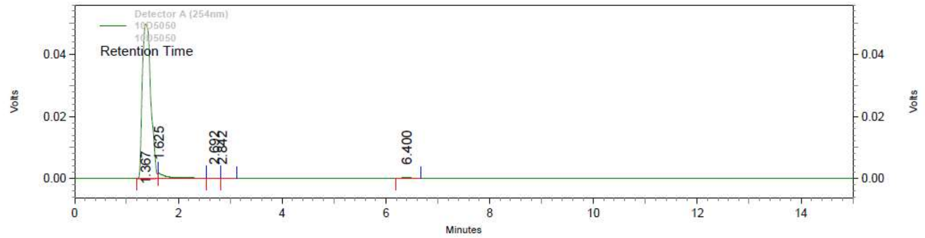

Detector A (254nm)

Pk #	Retention Time	Area	Area %	Height	Height %
1	1.367	566148	95.684	49791	96.241
2	1.625	21737	3.674	1613	3.118
3	2.692	1269	0.214	94	0.182
4	2.842	517	0.087	61	0.118
5	6.400	2016	0.341	177	0.342
Totals		591687	100.000	51736	100.000

HPLC compound 3e

Shimadzu CLASS-VP V6.14 SP2 *Area % Report*
Page 1 of 1

Method Name: C:\CLASS-VP\Methods\Polyphenols_MA_02.met
Data Name: C:\CLASS-VP\Data\Joaquin_Fernandez\Tito\10E50502
User: Mario
Acquired: 27/04/2022 16:46:11
Printed: 27/04/2022 17:25:26

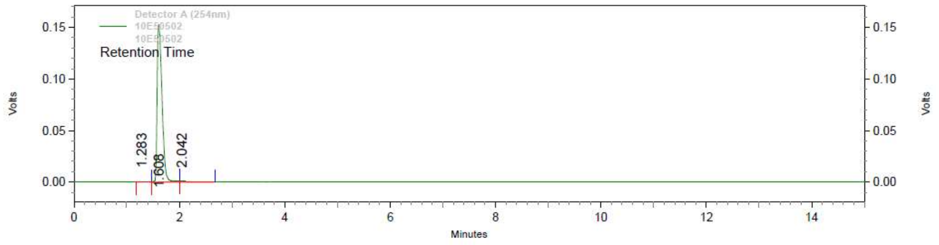

Detector A (254nm)

Pk #	Retention Time	Area	Area %	Height	Height %
1	1.283	5816	0.582	569	0.369
2	1.608	981867	98.261	152566	99.066
3	2.042	11556	1.156	869	0.564
Totals		999239	100.000	154004	100.000

HPLC compound 3f

Shimadzu CLASS-VP V6.14 SP2 *Area % Report*
Page 1 of 1

Method Name: C:\CLASS-VP\Methods\Polyphenols_MA_02.met
Data Name: C:\CLASS-VP\Data\Joaquin_Fernandez\Tito\10F50502
User: Mario
Acquired: 27/04/2022 17:28:14
Printed: 27/04/2022 19:00:00

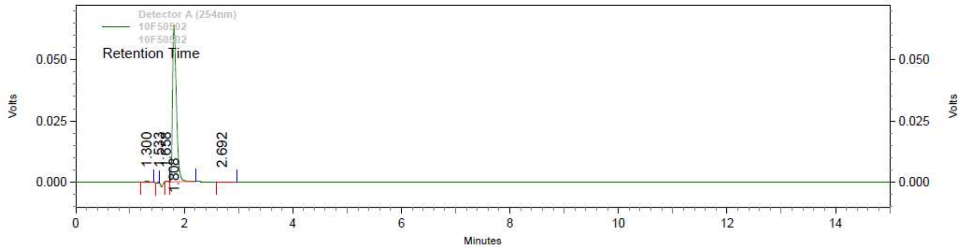

Detector A (254nm)

Pk #	Retention Time	Area	Area %	Height	Height %
1	1.300	1672	0.517	197	0.304
2	1.533	985	0.304	482	0.743
3	1.658	398	0.123	144	0.222
4	1.808	320177	98.962	64044	98.678
5	2.692	302	0.093	35	0.054
Totals		323534	100.000	64902	100.000

HPLC compound 3g

Shimadzu CLASS-VP V6.14 SP2 *Area % Report*

Page 1 of 1

Method Name: C:\CLASS-VP\Methods\Polyphenols_MA_02.met
Data Name: C:\CLASS-VP\Data\Joaquin_Fernandez\Tito\10G5050
User: Mario
Acquired: 27/04/2022 17:48:54
Printed: 27/04/2022 18:14:41

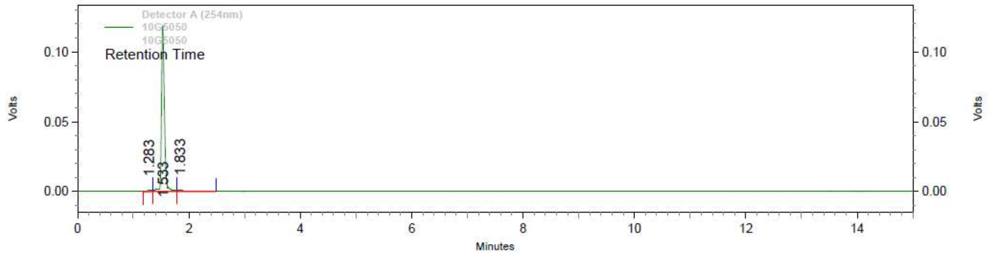

Detector A (254nm)

Pk #	Retention Time	Area	Area %	Height	Height %
1	1.283	4203	0.933	651	0.540
2	1.533	435091	96.588	118814	98.466
3	1.833	11165	2.479	1200	0.994
Totals		450459	100.000	120665	100.000

HPLC compound 3h

Shimadzu CLASS-VP V6.14 SP2 *Area % Report*

Page 1 of 1

Method Name: C:\CLASS-VP\Methods\Polyphenols_MA_02.met
Data Name: C:\CLASS-VP\Data\Joaquin_Fernandez\Tito\10H5050
User: Mario
Acquired: 27/04/2022 18:08:09
Printed: 27/04/2022 19:02:45

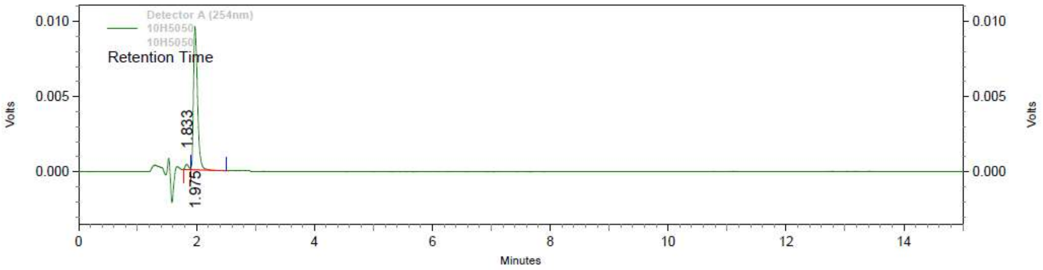

Detector A (254nm)

Pk #	Retention Time	Area	Area %	Height	Height %
1	1.833	1436	3.065	350	3.539
2	1.975	45418	96.935	9541	96.461
Totals		46854	100.000	9891	100.000

S1. Histamine and H_3R

The simulation of the histamine and H_3R was carried out by superimposing the 3RZE (inactive conformation) and 7DFL (active conformation) crystals. Subsequently, the ligand is removed from the 3RZE crystal and the receptor from the 7DFL crystal. As a result, the new model depicts the arrangement of the amino acids Asp114 and Glu206 in relation to histamine's primary amine and imidazole orientation.

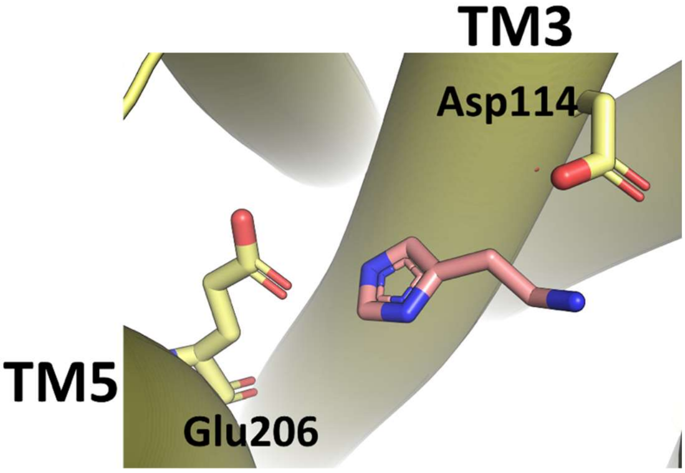

Figure S1. Graphical representation of histamine at the H_3R binding site.

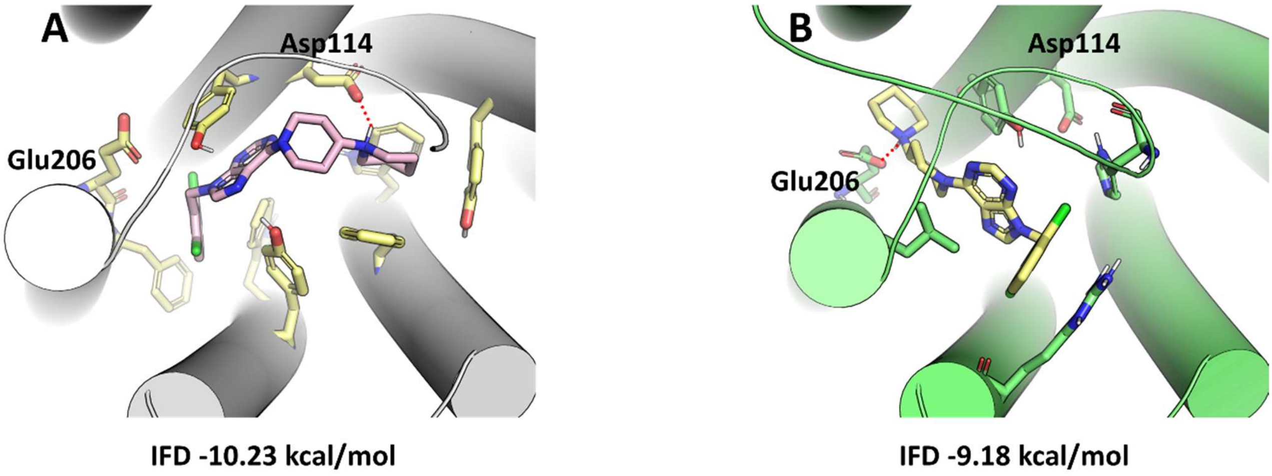

Figure S2. The two best poses were achieved with the induced-fit docking protocol for **3d** at the H3R binding site. (**A**) Piperidine oriented toward Asp114, and (**B**) piperidine oriented toward Glu206.

S2. Molecular dynamics analysis

S2.1. RMSD of systems **3a** and **3d**

The Root Mean Square Deviation (RMSD) is a metric for calculating a group of atoms' average change in displacement concerning a reference frame. It is calculated for each frame of the trajectory. For frame *x*, the RMSD is:

$$RMSD = \sqrt{\frac{1}{N}\sum_{i=1}^{N}\left(r_i'(t_x)\right) - \left(r_i(t_{ref})\right)^2}$$

where N is the number of atoms in the atom selection; t_{ref} is the reference time (typically the first frame is used as the reference, and it is regarded as time $t = 0$); and r' is the position of the selected atoms in frame x after superimposing on the reference frame, where frame x is recorded at time t_x. The procedure is repeated for every frame in the simulation trajectory.

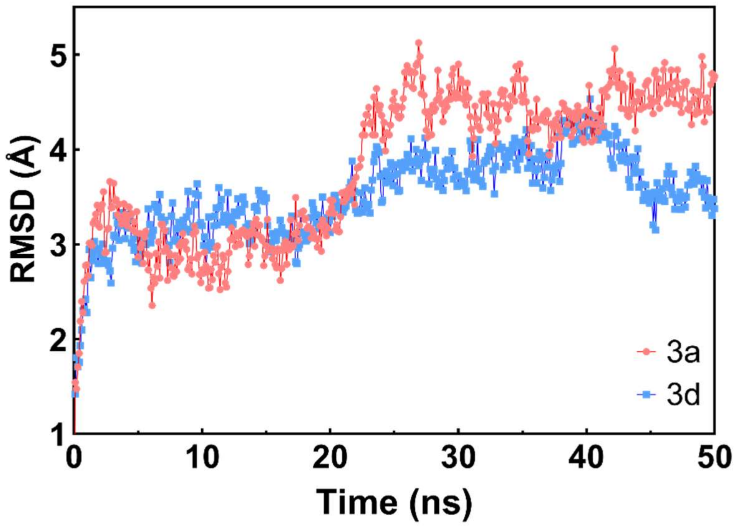

Figure S3. The RMSD of the two studied complexes (**3a** in red and **3d** in blue) was obtained during 50 ns of MD simulations.

The above plot shows the RMSD evolution of a protein (left Y-axis) in angstrom (Å). All protein frames are first aligned on the reference frame Cα of the protein, and then the RMSD is calculated based on the atom selection.

Monitoring the RMSD of the protein can give insights into its structural conformation throughout the simulation. RMSD analysis can indicate if the simulation has equilibrated; its fluctuations towards the end of the simulation are around some thermal average structure. Changes of the order of 1-3 Å are perfectly acceptable for small, globular proteins. However, more extensive changes indicate that the protein undergoes a significant conformational change during the simulation. For example, the H3R-3a system increases its RMSD from 20 ns onwards, the cause of which lies in the displacement of 3a at the binding site due to the low affinity achieved for the halogenated aromatic fragment of the hydrophobic pocket 2 (HP2). In contrast, the H3R-3d system has a better affinity for HP2, achieving a more stable RMSD (~ 3.5 Å).

S2.2. RMSF of systems **3a** and **3d**

The Root Mean Square Fluctuation (RMSF) is useful for characterizing local changes along the protein chain. The RMSF for residue *i* is:

$$RMSF_i = \sqrt{\frac{1}{T}\sum_{t=1}^{T} \langle \left(r_i'(t)\right)^2 - \left(r_i(t_{ref})\right)^2 \rangle}$$

where *T* is the trajectory time over which the RMSF is calculated, tref is the reference time, r_i is the position of residue *i*; r' is the position of atoms in residue *i* after superposition on the reference, and the angle brackets indicate that the average of the square distance is taken over the selection of atoms in the residue. On this plot, peaks indicate areas of the protein that fluctuate the most during the simulation.

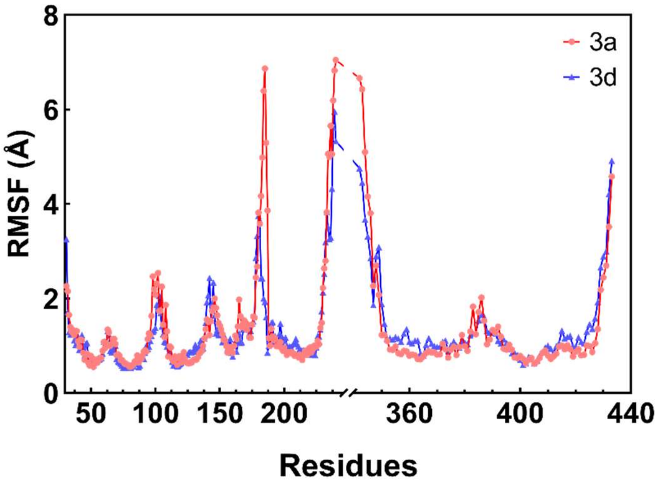

Figure S4. The root means square deviation (RMSF) values of each residue averaged over two trajectories for **3a** (red line) and **3d** (blue line).

S2.3. Ligand-protein contacts

Protein interactions with the ligand can be monitored throughout the simulation. As shown in the plot below, these interactions can be categorized by type and summarized. Protein-ligand interactions (or 'contacts') are categorized into four types: Hydrogen Bonds, Hydrophobic, Ionic, and Water Bridges. The stacked bar charts are normalized throughout the trajectory: for example, a value of 0.7 suggests that the specific interaction is maintained 70% of the simulation time. The threshold was 0.3 (dot line in red of Figure **S5-A** and **S6-A**) residue interactions with the respective ligand.

In Figure **S5-B** and **S6-B**, the top panel shows the total number of specific contacts the protein makes with the ligand for the trajectory (blue graphic). The bottom panel shows which residues interact with the ligand in each trajectory frame. Some residues make more than one specific contact with the ligand, which is represented by a darker shade of orange, according to the scale to the right of the plot.

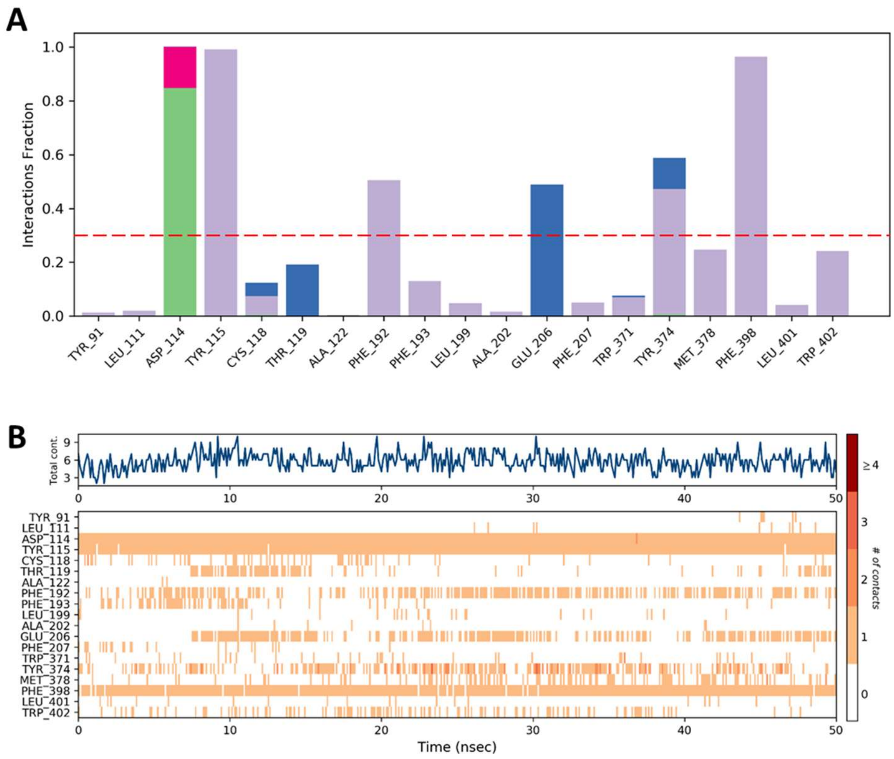

Figure S5. (**A**) Histogram plot of the contact H3R-**3a** system. (**B**) Timeline plots represent of the interactions and contact with **3a**.

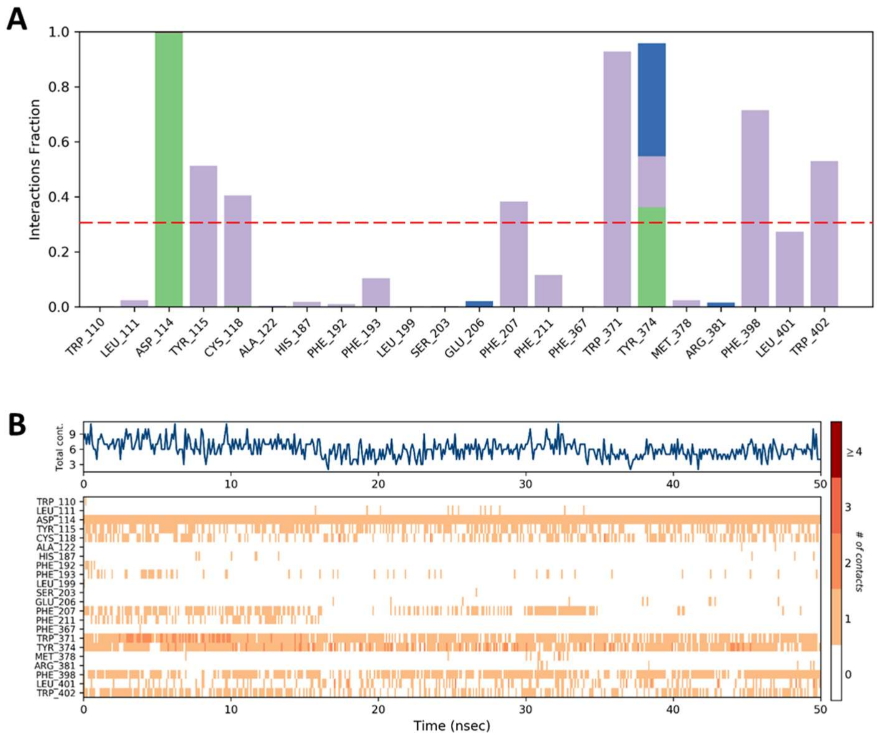

Figure S6. (**A**) Histogram plot of the contact H3R-**3d** system. (**B**) Timeline plots represent of the interactions and contact with **3d**.

According to the H3R-3a system are Asp114, Phe192, Glu206, Tyr374, and Phe398, the 0.3 threshold and only Asp114, Tyr115, and Phe378 exceed 80% during molecular dynamics. In contrast, the H3R-**3d** system has Asp114, Tyr115, Cys118, Trp371, Tyr374, Phe398, Trp402, exceeding the 0.3 thresholds; however, the amino acids Asp114, Trp371, and Tyr374 exceed 80% during molecular dynamics, a pattern of interactions similar to that achieved with pitolisant (10.1111/cbdd.13471)

S2.4. Ligand properties in the binding site

The properties of the ligand give a clear idea about the global interactions affecting the ligand at the binding site, such as the evolution of its RMSD, the radius of gyration, Molecular surface area, Solvent accessible surface area and Polar Surface area, whose properties are defined as:

Ligand RMSD: Root mean square deviation of a ligand with respect to the reference conformation (typically, the first frame is used as the reference, and it is regarded as time t=0).

The radius of Gyration (rGyr): Measures the 'extendedness' of a ligand and is equivalent to its principal moment of inertia.

Molecular Surface Area (MolSA): Molecular surface calculation with 1.4 Å probe radius. This value is

equivalent to a van der Waals surface area.

Solvent Accessible Surface Area (SASA): Surface area of a molecule accessible by a water molecule.

Polar Surface Area (PSA): Solvent accessible surface area in a molecule contributed only by oxygen and nitrogen atoms.

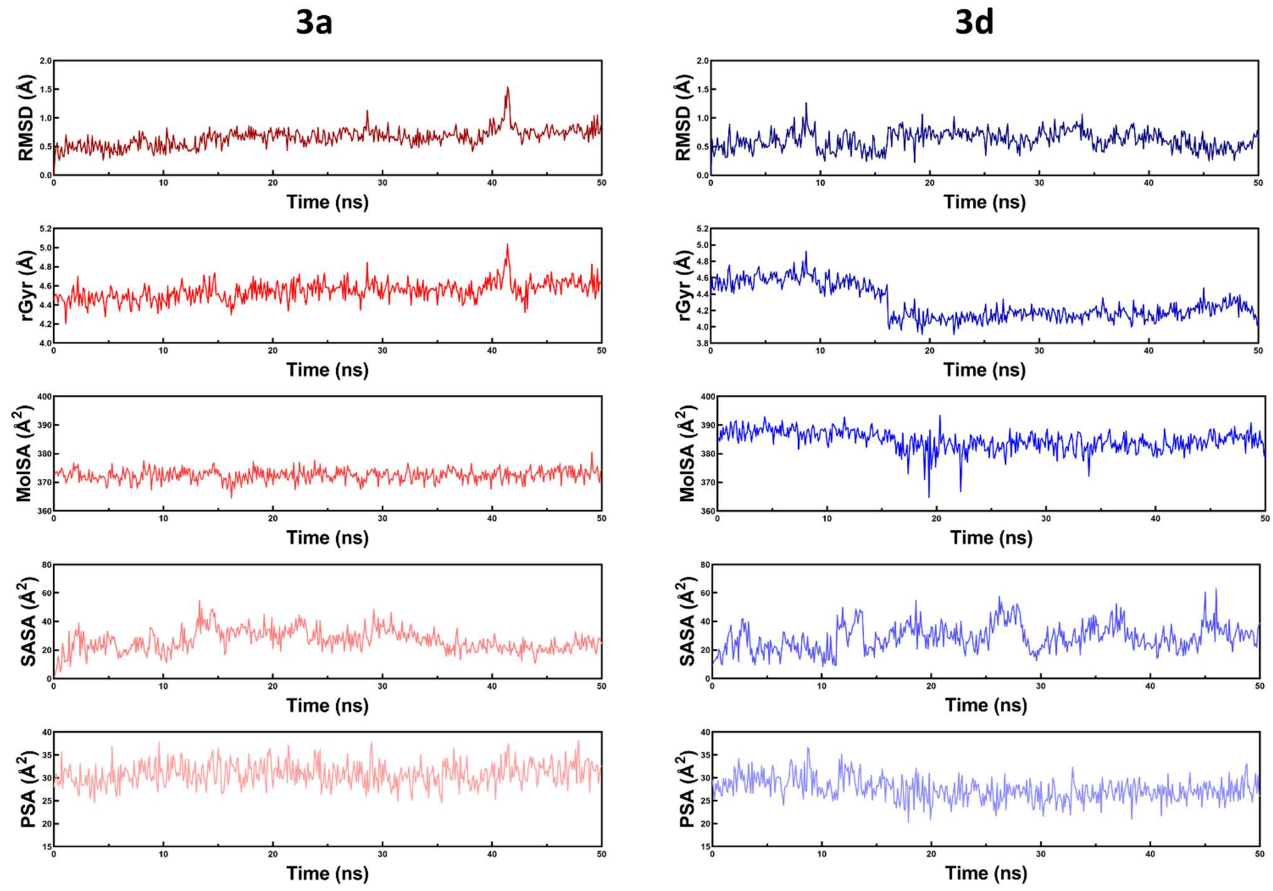

Figure S7. Graphical representations of **3a** and **3d** properties in the binding site.
